# The mutualism effector MiSSP7 of *Laccaria bicolor* alters the interactions between the poplar JAZ6 protein and its associated proteins

**DOI:** 10.1038/s41598-020-76832-6

**Published:** 2020-11-23

**Authors:** Yohann Daguerre, Veronica Basso, Sebastian Hartmann-Wittulski, Romain Schellenberger, Laura Meyer, Justine Bailly, Annegret Kohler, Jonathan M. Plett, Francis Martin, Claire Veneault-Fourrey

**Affiliations:** 1 UMR 1136, Interactions Arbres/Microorganismes (IAM), Centre INRAE de Nancy, Université de Lorraine/INRAE, Champenoux, France; 2 Present Address: Hawkesbury Institute for the Environment, Western Sydney University, Penrith, NSW 2751 Australia; 3 Present Address: Department of Forest Genetics and Plant Physiology, Umeå Plant Science Centre, Swedish University of Agricultural Sciences, 901 83 Umeå, Sweden

**Keywords:** Plant symbiosis, Fungi

## Abstract

Despite the pivotal role of jasmonic acid in the outcome of plant-microorganism interactions, JA-signaling components in roots of perennial trees like western balsam poplar (*Populus trichocarpa*) are poorly characterized. Here we decipher the poplar-root JA-perception complex centered on PtJAZ6, a co-repressor of JA-signaling targeted by the effector protein MiSSP7 from the ectomycorrhizal basidiomycete *Laccaria bicolor* during symbiotic development. Through protein–protein interaction studies in yeast we determined the poplar root proteins interacting with PtJAZ6. Moreover, we assessed via yeast triple-hybrid how the mutualistic effector MiSSP7 reshapes the association between PtJAZ6 and its partner proteins. In the absence of the symbiotic effector, PtJAZ6 interacts with the transcription factors PtMYC2s and PtJAM1.1. In addition, PtJAZ6 interacts with it-self and with other *Populus* JAZ proteins. Finally, MiSSP7 strengthens the binding of PtJAZ6 to PtMYC2.1 and antagonizes PtJAZ6 homo-/heterodimerization. We conclude that a symbiotic effector secreted by a mutualistic fungus may promote the symbiotic interaction through altered dynamics of a JA-signaling-associated protein–protein interaction network, maintaining the repression of PtMYC2.1-regulated genes.

## Introduction

As an adaptation to nitrogen- and phosphorous -limiting conditions, the root systems of forest trees interact with the hyphae of mutualistic ectomycorrhizal (ECM) fungi to form a hybrid organ called the ectomycorrhizal root tip. To build this tissue, ECM fungal hyphae first grow towards lateral roots and aggregate on the root surface forming a hyphal sheath called the mantle around root tips. In a second step, fungal hyphae grow within the apoplastic space of root cortical cells to form the biotrophic interface named the Hartig net. This interface is the site of bi-directional nutrient exchanges between root cells and fungal cells. Here, water and growth-limiting nutrients are sourced by the soil-borne extraradical mycelium and exchanged with photosynthetic carbon from the host tree^1,2^. In addition to improving tree nutrition, ECM fungi stimulate the plant immune system, leading to induced systemic resistance (ISR) against root and shoot fungal pathogens and aboveground herbivores^3,4^. Furthermore, ECM fungi enhance tree tolerance to salinity, drought and heavy metals^5–9^. Despite the crucial importance of ECM fungi for the nutrition and health of trees, the identification of master regulators involved in the molecular dialogue between plant and fungal cells is still in its infancy. Indeed, the establishment, development and maintenance of functional ectomycorrhizal root tips require a strict coordination of the development, immunity and physiology of both fungal and plant cells. These processes are likely to be regulated, at least in part, by phytohormones. Exploiting the mutualistic interaction occurring between *Laccaria bicolor* and *Populus* spp. as a model to study ECM symbiosis at the molecular level, we have previously demonstrated that hyphae of the fungus *L. bicolor* secrete Mycorrhiza-induced Small Secreted Proteins (MiSSPs) in the vicinity of poplar roots^10^. In particular, MiSSP7, a symbiotic effector required for ECM development^11^, hinders jasmonate (JA)-mediated responses in poplar root cells^12^ . To do so, MiSSP7 physically interacts with the *Populus* JAZ6 (PtJAZ6) protein in nuclei of host cells. This interaction inhibits the degradation of PtJAZ6 in the presence of JA and consequently suppresses JA-dependent responses allowing *in planta* fungal colonization. In addition, exogenous treatment with MeJA inhibited the development of ectomycorrhizal root tips; in particular the formation of the Hartig net^12^. Altogether, previous studies highlight that the mutualistic fungus *L. bicolor* hijacks the host JA-signaling pathway, but in an opposite manner as compared to biotrophic plant-pathogenic microbes^13,14^.

JAs are well-known plant defense hormones involved in responses to wounding and herbivory^15^. Furthermore, JAs regulate plant developmental processes such as root development^16^, fertility^17^, senescence^18^, secondary metabolites production^19^ and response to abiotic stress^20–22^. During the last decade, considerable effort has been devoted to elucidate the JA-signaling pathway, JA-triggered responses, and how the JA-signaling pathway is integrated with other hormone-mediated responses^23,24^. However, the majority of these studies on JA pathways have been performed on the leaves of *Arabidopsis*, tomato, or rice.

In these well-studied model systems, the bioactive JA-hormone (i.e., (+)-7-iso-jasmonoyl-L-Ile)^25^, is perceived by a co-receptor complex consisting of dimerized JASMONATE ZIM-DOMAIN (JAZ) proteins, which form part of a transcription factor (TF) repression complex, and the F-box protein CORONATIN INSENSITIVE1 (COI1), which is part of a Skp1/Cullin/F-box E3 ubiquitin ligase^26^. JA perception leads to ubiquitination of the JAZ proteins, tagging them for 26S-proteasome-mediated degradation, thereby releasing inhibition of downstream TFs and initiating the activation of JA-dependent responses. Therefore, members of the JAZ family are key repressors of JA-mediated hormone responses. JAZ proteins interact with a wide range of transcription factors (TFs) and are thus major hubs integrating environmental and developmental cues in order to modulate several developmental and physiological processes on the basis of a growth-to-defense energetic trade-off^23,27,28^.

The *JAZ* family in *Arabidopsis* is composed of 12 canonical members and the atypical *JAZ13* repressor^29^. Canonical JAZ proteins show three structural domains mediating protein–protein interactions: a variable N-terminal (NT) domain, a highly conserved ZIM domain and a conserved C-terminal JA-associated (Jas) domain. This last domain is required for binding to COI1 and TFs^30,31^. The central ZIM domain contains a highly conserved TIFY motif (TIF(F/Y)XG) required for homo- and heterodimerization of JAZ proteins as well as for the recruitment of TOPLESS (TPL) or TOPLESS-RELATED (TPR) transcriptional repressors, either directly bound through an ETHYLENE RESPONSIVE FACTORS-associated amphiphilic repression (EAR) motif or indirectly bound through the NOVEL INTERACTOR OF JAZ (NINJA) bridge protein^31–33^.

In *Arabidopsis* JAZ-interacting TFs include both transcriptional activators and repressors modulating several developmental and physiological processes, such as suppression of root elongation, biosynthesis of secondary metabolites, trichome development and pollen maturation^23,24^. JA-signaling activators include basic helix-loop-helix (bHLH) proteins such as MYC2 which are involved in plant defense, glucosinolates biosynthesis, pollen development and growth inhibition^18,23,34,35^. JAZ proteins also interact with the transcriptional repressors JAM1/2/3 and bHLH14 which probably inhibit excessive defense responses to avoid stunted growth^17,36–38^.

Most of current knowledge on JA-signaling and its related proteins was obtained from the model plant *Arabidopsis.* The diversity of JA-dependent responses in other organs such as roots or in perennial plants, however, is less well understood^39,40^. More importantly, to our knowledge, there are no detailed reports of JA-signaling components in roots of perennial trees that form mycorrhizal interactions within their root systems. This latter point emphasizes a critical gap in our knowledge of this pathway, as plant-associated organisms are known to manipulate JA-Ile signaling through the use of effectors, which modify JAZ stability^12,41–44^, interfere with the activity of JA-regulated TFs^44,45^, or influence JA-Ile biosynthesis^30,46–48^. In addition, even if it has been demonstrated that the secreted symbiotic effector MiSSP7 is fundamental for PtJAZ6 stabilization and for the development of ectomycorrhiza between *Populus* and the basidiomycete *L. bicolor*^12^, the consequences of such PtJAZ6 stabilization on poplar root regulatory networks are still unexplored. Therefore, in the present paper we aimed to decipher the identity and the dynamics of the association between PtJAZ6 and its partner proteins in poplar ECM roots. We intended to answer the three following questions: Which poplar proteins are interacting with PtJAZ6 in poplar root cells? Which PtJAZ6 domains are required for such interactions? How does the stabilization of PtJAZ6 by the symbiotic effector MiSSP7 impact the strength of poplar protein–protein interactions?

## Results

### The transcription factors PtJAM1 and PtMYC2 proteins interact with PtJAZ6

**Figure 1:**
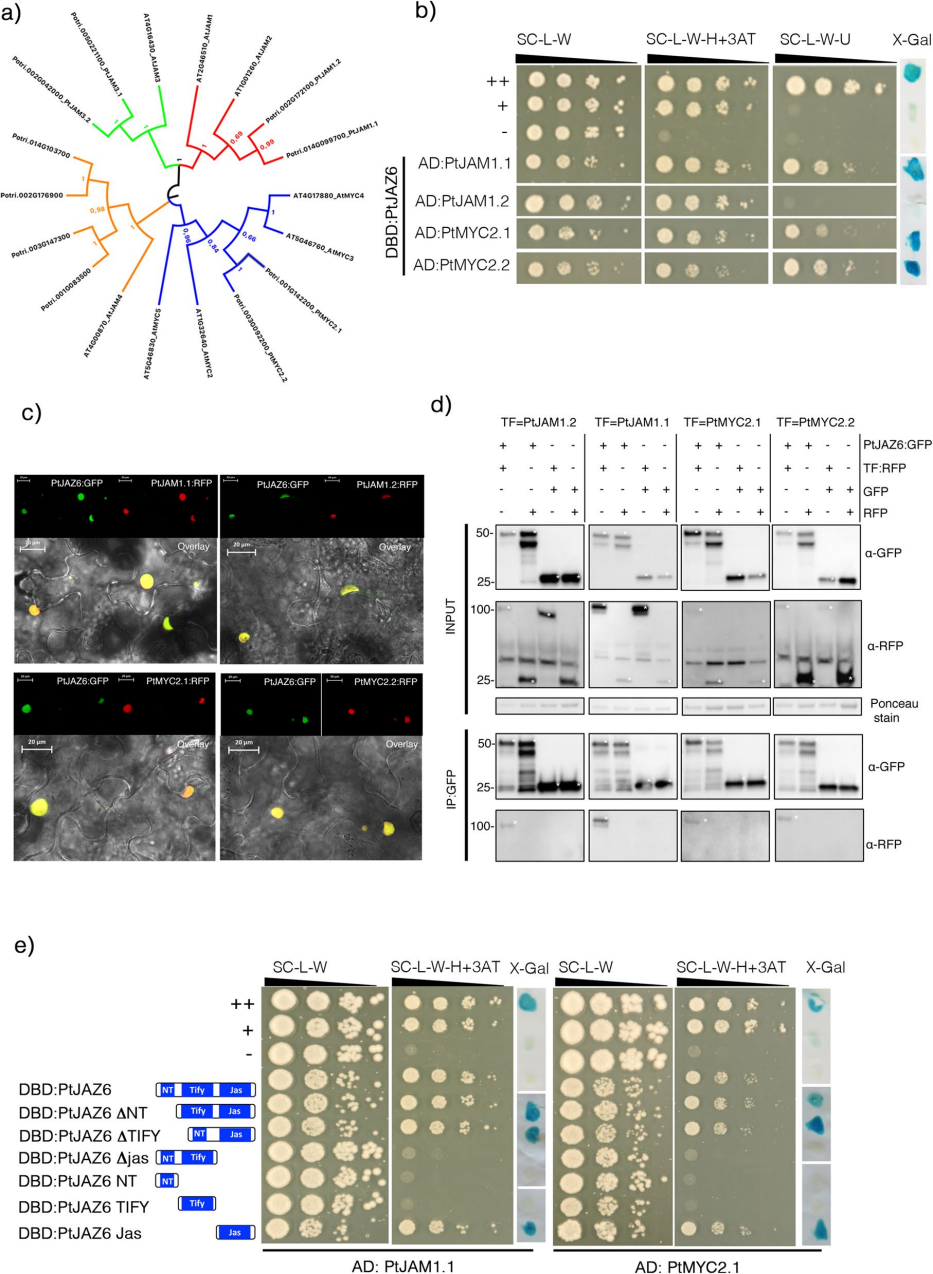
PtJAZ6 interacts with PtJAM1 and PtMYC2 transcription factors in yeast and *in planta*. (**a**) Phylogenetic analysis of the bHLH transcription factors orthologous to *Arabidopis thaliana* MYCs and JAMs. The evolutionary history was inferred through the Maximum Likelihood method and conducted in MEGA7. The values of the 1000 performed bootstraps are indicated. FigTree v1.4.4 (https://tree.bio.ed.ac.uk/software/figtree/) was used to draw the tree. (**b**) Yeast-two hybrid (Y2H) assays to reveal interactions of PtJAZ6 with PtJAM1.1, PtJAM1.2, PtMYC2.1 or PtMYC2.2. PtJAZ6 was fused to the GAL4 DNA-binding domain (DBD) whereas the various transcription factors (TFs) were fused to the GAL4-activation domain (AD). *TRP1* and *LEU2* served as transformation markers for vectors whereas *HIS3*, *URA3* and β*-galactosidase LacZ* (X-Gal) were used as reporter genes. (**c**) Live-cell imaging of PtJAZ6:GFP and TF:RFP transiently expressed in *Nicotiana benthamiana* epidermal leaf cells transformed by agroinfiltration, using a laser-scanning confocal microscope in a sequential scanning mode. Scale bar: 20 μm. (**d**) *In planta* co-immunoprecipitation of PtJAZ6 with PtJAM1 or PtMYC2 proteins. Fusion proteins (PtJAZ6:GFP and TF:RFP) were isolated from agroinfiltrated leaves. Both initial proteins (Input) and immunoprecipitated protein (IP-GFP) were separated by 12% SDS-PAGE and electro-transferred onto PVDF membranes. Immunodetection was performed with anti-GFP or anti-RFP antibodies, and immunoblots were revealed with a chemiluminescent imager. Ponceau staining of the PVDF membrane was used as a loading control. White asterisks indicate specific protein bands. Numbers on the left side of the blots indicate protein size in kilodaltons. (**e**) Y2H assays to assess which domains of PtJAZ6 are required for its interaction with PtMYC2.1 and PtJAM1.1. PtMYC2.1 and PtJAM1.1 were fused to the GAL4 AD, whereas full-length or mutated versions of PtJAZ6 were fused with the GAL4 DBD. *TRP1* and *LEU2* served as transformation markers for vectors whereas *HIS3* and β*-galactosidase LacZ* (X-Gal) were used as reporter genes. For Y2H (**b** and **e**): 3-Amino-1, 2, 4-triazole (3-AT) was used to suppress self-activation of the *HIS3* gene. The control colonies are shown in the upper part of the panel: ++ is a strong positive interaction, + is a weakly interacting protein pair, — is a negative control (no interaction). Black and white lines indicate cropped and repositioned images while the full-length blots and gels are presented in Supplementary Information online.

In order to identify PtJAZ6-interacting proteins, we conducted a yeast two-hybrid (Y2H) screen. The full-length coding sequence of PtJAZ6 was cloned in frame with the DNA-binding domain (DBD) of GAL4. We first confirmed that this fusion protein is not able to activate the three reporter genes in the absence of an interacting protein (Supplementary Figure S1). Among positive interactors, we identified PtJAZ6 (Potri.003G068900), MiSSP7 and a basic-helix-loop-helix (bHLH) transcription factors similar to *Arabidopsis* JAM1/2 (orthologous to At1g01260 and At2g46510), that we thus named PtJAM1.1. As the *Populus* genome contains two co-orthologous genes to *AtJAM1*(Fig. 1a), we cloned the full-length sequence of the two paralogs *PtJAM1.1* (Potri.014G099700.1) and *PtJAM1.2* (Potri.002G172100.1) fused with the activation domain (AD) of *GAL4* and co-expressed them with *PtJAZ6* fused to the DBD of *GAL4*. In *Arabidopsis* AtJAZ proteins are known to interact with AtJAMs transcriptional repressors as well as with the transcriptional activators AtMYC2-5^49^. In the *Populus* genome, we found only two orthologous genes to *AtMYC2-5.* We named them *PtMYC2.1* (Potri.001G142200) and *PtMYC2.2* (Potri.003G092200) and we cloned their full-length sequences fused to the AD of GAL4. We tested the expression of three reporter genes to assess the strength of protein–protein interactions. Growth of the transformed yeasts on selective media lacking uracil and development of a dark blue color in the X-gal assay indicated a strong interaction, while weak interactions were detected by the absence of growth on medium lacking uracil but growth on medium lacking histidine, in addition to a faint or absent coloration in the X-gal assay. Finally, non-interacting protein pairs were identified by the absence of growth on medium lacking histidine. Through these assays, we demonstrated that the two PtMYC2 and PtJAM1.1 protein strongly interact with PtJAZ6 in yeasts, whereas PtJAM1.2 only weakly interacts with PtJAZ6 (Fig. 1b).

To validate these interactions *in planta,* and to determine the sub-cellular localization of these proteins, we transiently co-expressed *PtMYC2s:RFP/PtJAZ6:GFP* or *PtJAM1s:RFP/PtJAZ6:GFP* in tobacco leaves and performed co-immunoprecipitation and confocal microscopy. Transient expression of these constructs in tobacco epidermal cells indicated that PtJAZ6:GFP, PtMYC2s:RFP and PtJAM1s:RFP fusion proteins accumulated in nuclei of *Nicotiana benthamiana* leaf epidermal cells (Fig. 1c). Moreover, proteins were extracted from the agroinfiltrated leaves and both fusion proteins were detected by Western blots (Fig. 1d). The CoIP experiments revealed that PtMYC2s:RFP and PtJAM1s:RFP associated with PtJAZ6:GFP (Fig. 1d). Neither free GFP nor free RFP were able to immunoprecipitate PtMYC2s/PtJAM1s:RFP or PtJAZ6:GFP, respectively, indicating that the associations between PtMYC2s and PtJAZ6 or PtJAM1s and PtJAZ6 are specific to the proteins of interest and not due to interactions between the fluorophore tags (Fig. 1d). We conclude that PtMYC2s and PtJAZ6 as well as PtJAM1s and PtJAZ6, physically interact within plant nuclei. However, since PtJAM1.2 interacted with PtJAZ6 only weakly, we decided to exclude it from further analysis.

Since poplar JAM1 and MYC2 proteins exhibit between 33 to 38% sequence identity and nearly identical bHLH domains with respect to their *Arabidopsis* counterparts (Supplementary Figure S2a,b), these proteins are likely TFs. To test this hypothesis, we assessed via a yeast one-hybrid assay, whether PtMYC2.1, PtMYC2.2 or PtJAM1.1 fused to the DBD of GAL4 could induce the expression of the *HIS3* and *LacZ* reporter genes. We found that expression of DBD:PtMYC2.1 or DBD:PtMYC2.2 activated the two reporter genes, whereas the expression of DBD:PtJAM1.1 did not (Supplementary Figure S2c). Our data suggest that PtMYC2s are transcriptional activators while PtJAM1.1, similar to its *Arabidopsis* homologs, might be a transcriptional repressor.

### The PtJAZ6 Jas domain is required and sufficient for the interaction with PtJAM1.1 and PtMYC2.1

PtJAZ6 contains a variable N-terminal (NT) domain, a central ZIM domain (TIFY) and a C-terminal Jas domain (Supplementary Figure S3). To dissect the role of the different domains in the interactions of the PtJAZ6 with its partner proteins, we constructed different deletion mutants of PtJAZ6, namely PtJAZ6 ΔNT, PtJAZ6 ΔTIFY, and PtJAZ6 ΔJas, deleted respectively for the NT, TIFY or Jas domains. We also made constructions harboring a single domain and named them: PtJAZ6 NT, PtJAZ6 TIFY and PtJAZ6 Jas. All these constructions were fused to GAL4 DBD and expressed in yeast strains harboring different members of the PtJAZ6-associated proteins to the GAL4 AD. Yeast growth on selective medium lacking histidine showed that PtJAM1.1 and PtMYC2.1 could not interact with versions of PtJAZ6 lacking the Jas domain, while they could interact with a version of PtJAZ6 containing the Jas domain only (Fig. 1e and Supplementary Figure S4a). We conclude that the Jas domain of PtJAZ6 is necessary and sufficient for the interaction between PtJAZ6 and the transcription factors PtMYC2.1 and PtJAM1.1.

### PtJAZ6 physically interacts with other poplar JAZ proteins

**Figure 2:**
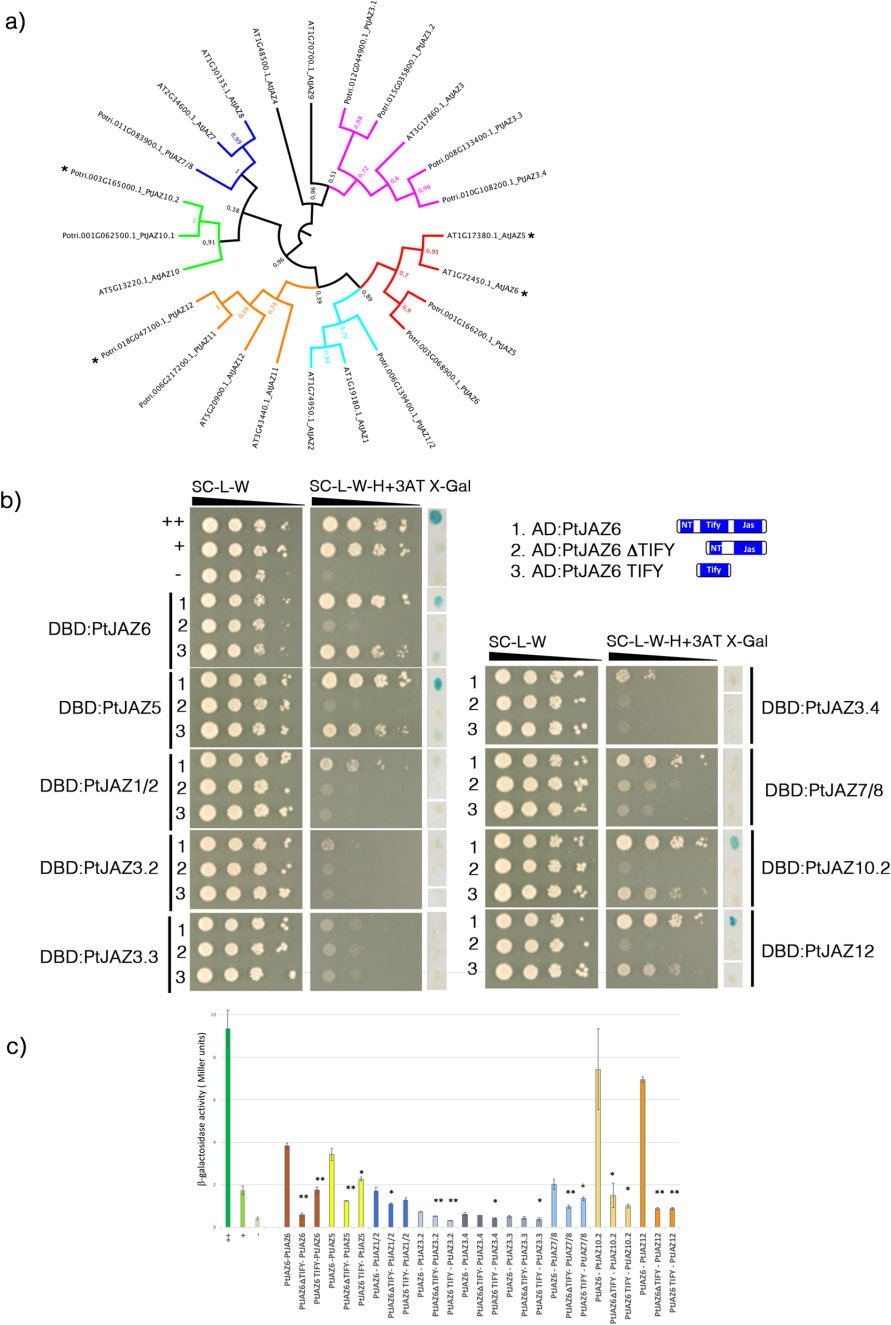
PtJAZ6 can form homodimers and heterodimers with PtJAZ proteins through its TIFY domain. (**a**) Phylogenetic tree of canonical JAZ proteins from *Arabidopsis* and *Populus*. The evolutionary history was inferred through the Maximum Likelihood method and conducted in MEGA7. The values of the 1000 performed bootstraps are indicated. *Indicate PtJAZ proteins interacting with PtJAZ6 forming homo- and heterodimers. FigTree v1.4.4 (https://tree.bio.ed.ac.uk/software/figtree/) was used to draw the tree. (**b**) Yeast-two hybrid (Y2H) assays to detect interactions of PtJAZ6 with itself and with other PtJAZ proteins expressed in ECM root tips. PtJAZ proteins were fused to the GAL4 DBD. PtJAZ6 or PtJAZ6 deleted for its TIFY domain (PtJAZ6ΔTIFY) or containing the TIFY domain only (PtJAZ6 TIFY) were fused with the GAL4 AD. *TRP1* and *LEU2* served as transformation markers for vectors whereas *HIS3* and β*-galactosidase LacZ* (X-Gal) were used as reporter genes. The control colonies are shown in the upper part of the panel: ++ is a strong positive interaction, + is a weakly interacting pair, – is a negative control (no interaction). 3-Amino-1, 2, 4-triazole (3-AT) was used to suppress self-activation of the *HIS3* gene. White lines indicate cropped and repositioned images while the full-length blots and gels are presented in Supplementary Information online. (**c**) Quantification of β-galactosidase activity in yeast colonies showed in (b) through ONPG assay. ++ is a strong positive interaction, + is a weakly interacting pair, — is a negative control (no interaction). Significant differences between the interaction of PtJAZ proteins and the different deleted versions of PtJAZ6 were assessed via pairwise Student’s t-test (*p < 0.05, **p < 0.01).

To explore the possible interactions between PtJAZ6 and other PtJAZ proteins, we tested AD:PtJAZ6 and DBD:PtJAZs interaction in the yeast two-hybrid system described above. The *Populus* genome contains 12 canonical PtJAZ-encoding genes, the same number as in *Arabidopsis*. We classified and named them according to their phylogenetic position (Fig. 2a). We successfully cloned nine of these *PtJAZs* genes from a cDNA library of poplar-*L. bicolor* ECM root tips (Table S1). We then conducted yeast-two-hybrid (Y2H) studies using all pairs of candidate interactors to identify possible PtJAZ6 homo- or heterodimers. In addition, we exploited the PtJAZ6 ΔTIFY and the PtJAZ6 TIFY versions of PtJAZ6 to test whether the highly conserved TIF(F/Y)XG motif within the ZIM domain of PtJAZ6 (Supplementary Figure S3) is required for PtJAZ6 homo/heterodimerization. We found that PtJAZ6 could form homodimers with itself and heterodimers with PtJAZ5, PtJAZ10.2 and PtJAZ12 (Fig. 2b,c). According to a quantitative β-galactosidase assay, PtJAZ6 also interacted weakly with PtJAZ1.2 and PtJAZ7/8 (Fig. 2c). In all positive interactions, the deletion of the TIFY domain of PtJAZ6 inhibited the dimerization in the Y2H system (Fig. 2b,c). In addition, the TIFY domain of PtJAZ6 alone ensured PtJAZ6 homo- and heterodimerization with PtJAZ5 and PtJAZ1.2, but not with PtJAZ10.2, PtJAZ12, and PtJAZ7/8 (Fig. 2c). From our results we conclude that PtJAZ6 interacts with itself and the other JAZ proteins PtJAZ5, PtJAZ10.2 and PtJAZ12. We also show that the TIFY domain is required for the homodimerization of PtJAZ6 and its heterodimerization with PtJAZ5.

### PtJAZ6 interacts with two poplar NINJA proteins and one TOPLESS protein

**Figure 3:**
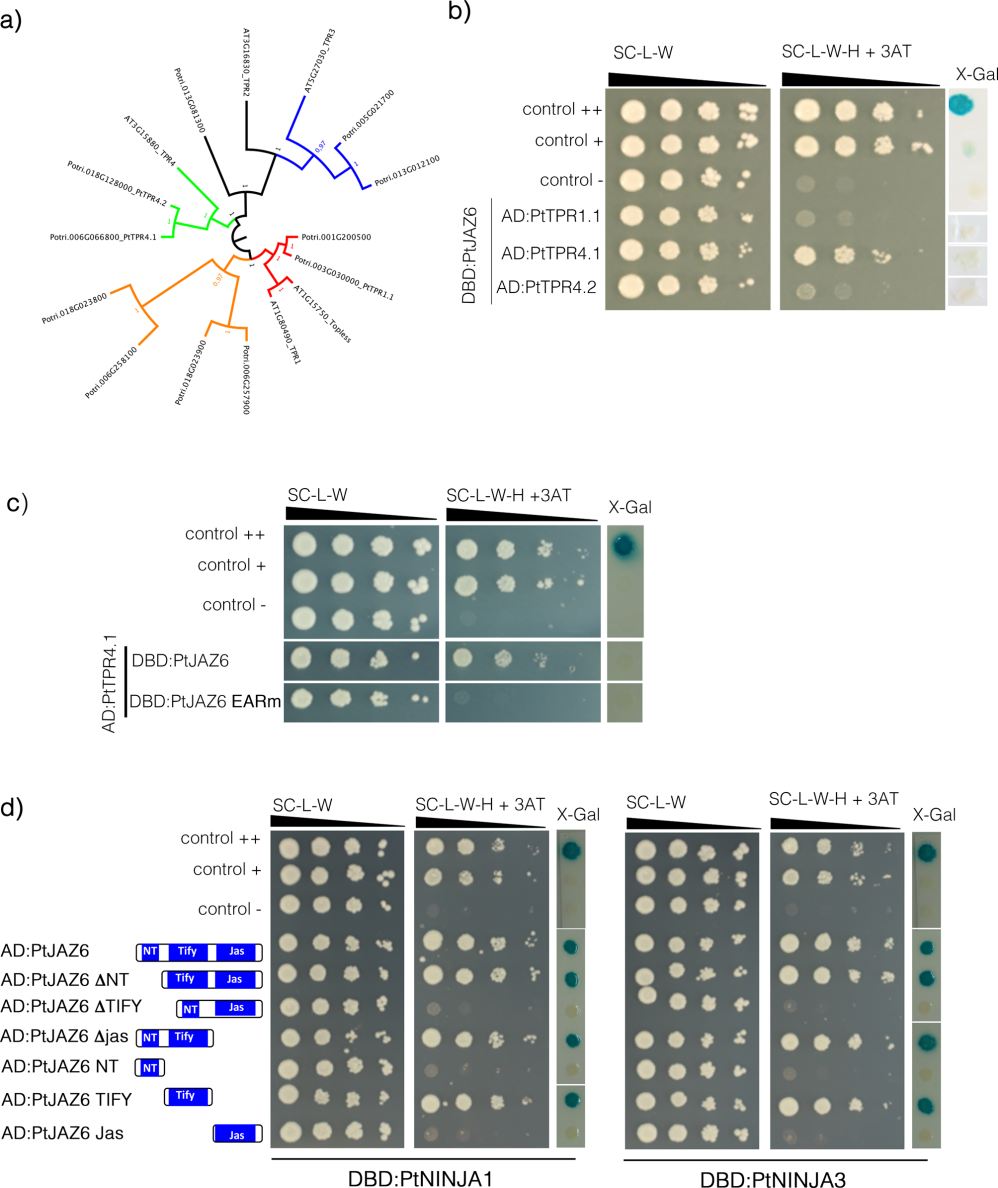
PtJAZ6 interacts with one PtTOPLESS-related protein and two NINJA proteins. (**a**) Phylogenetic tree of *Populus trichocarpa* TOPLESS-related proteins expressed in poplar roots. The evolutionary history was inferred by using the Maximum Likelihood method and conducted in MEGA7. The values of the 1000 performed bootstraps are indicated. The three poplar TOPLESS-related proteins cloned from a poplar ECM cDNA library are indicated as PtTPR1.1, PtTPR4.1, and PtTPR4.2. FigTree v1.4.4 was used to draw the tree. (**b**) Yeast-two hybrid (Y2H) assays to reveal interactions of PtJAZ6 with the TOPLESS-related protein PtTPR4.1 expressed in ECM root tips. PtJAZ6 was fused to GAL4 AD whereas the three TOPLESS-related proteins were fused to the GAL4 DBD. (**c**) The C-terminus EAR motif of PtJAZ6 is required for its interaction with PtTPR4.1 in yeasts. Yeast-two hybrid (Y2H) assays were performed with yeast co-expressing DBD:PtTPR4.1 and AD:PtJAZ6 or AD:PtJAZ6 mutated for its EAR motif (EARm). (**d**) PtJAZ6 interacts with PtNINJA1 and PtNINJ3 and the TIFY domain of PtJAZ6 is necessary and sufficient for the interaction. Y2H assays were performed with yeast co-expressing DBD:PtNINJA1 or DBD:PtNINJA3 and full length or mutated version of PtJAZ6 were fused with the GAL4 AD. For all yeast-two hybrid assays (b,c,d), *TRP1* and *LEU2* served as transformation markers for vectors and *HIS3* and β*-galactosidase LacZ* (X-Gal) were used as reporter genes. 3-Amino-1, 2, 4-triazole (3-AT) was used to suppress self-activation of the *HIS3* gene. The control colonies are shown in the upper part of the panel: ++ is a strong positive interaction, + is a weakly interacting pair, — is a negative control (no interaction). White lines indicate cropped and repositioned images while the full-length blots and gels are presented in Supplementary Information online.

*Arabidopsis* JAZ proteins form a repressor complex through the recruitment of the general repressor TOPLESS (TPL) and TPL-related (TPR) proteins either directly or indirectly through interaction with the bridge protein NINJA^31^. To first test the direct interaction between PtJAZ6 and TOPLESS, we successfully cloned three poplar *TPR* genes expressed in ECM root tips (*Potri.006G066800* alias *PtTPR4.1*; *Potri.018G128000* alias *PtTPR4.2* and *Potri.003G030000* alias *PtTPR1.1*) (Table S1; Fig. 3a). Exclusively PtTPR4.1 physically interacted with PtJAZ6 in yeast cells similar to the weak-interaction control (Fig. 3b). Since *Arabidopsis* TOPLESS-related proteins interact with the EAR motif found on some AtJAZ proteins^50,51^, we mutated the EAR motif located at the C-terminus of PtJAZ6 (Supplementary Figure S3). Yeasts co-expressing PtJAZ6 EARm and PtTPR4.could not grow on the selective medium (Fig. 3d), suggesting that the EAR motif of PtJAZ6 is required for its weak interaction with PtTPR4.1. In addition to the direct interaction of PtJAZ6 with PtTPR4.1, we tested whether PtJAZ6 is able to recruit any of the PtNINJA proteins. The *P. trichocarpa* genome contains three co-orthologues (*Potri.006G156100.1*; *Potri.006G162900.1*; *Potri.018G085100.1*) of the *Arabidopsis NINJA* gene (*AT4G28910.1*), all expressed in ECM root tips (Table S1). We were able to clone both *Potri.018G085100.1*, encoding PtNINJA3, and *Potri.006G162900.1*, encoding PtNINJA1 from a cDNA library of *L. bicolor*-poplar ECM root tips. PtJAZ6 physically interacted with both PtNINJA1 and PtNINJA3 in yeast cells (Fig. 3d). On the other hand, yeast growth and β-galactosidase activity assays showed that PtNINJA3 or PtNINJA1 could not interact with versions of PtJAZ6 lacking the TIFY domain, while they could interact with a version of PtJAZ6 containing the TIFY domain only (Fig. 3d and Supplementary Figure S4a). This indicates that the TIFY domain of PtJAZ6 is required and sufficient to mediate the interaction with PtNINJA3 and PtNINJA1 in yeast cells. From these data, we conclude that PtJAZ6 weakly interacts with the PtTPR4.1 TOPLESS-related protein and strongly interacts with two PtNINJA proteins.

### MiSSP7 physically interacts with PtJAZ6 through its NT and TIFY domains

**Figure 4:**
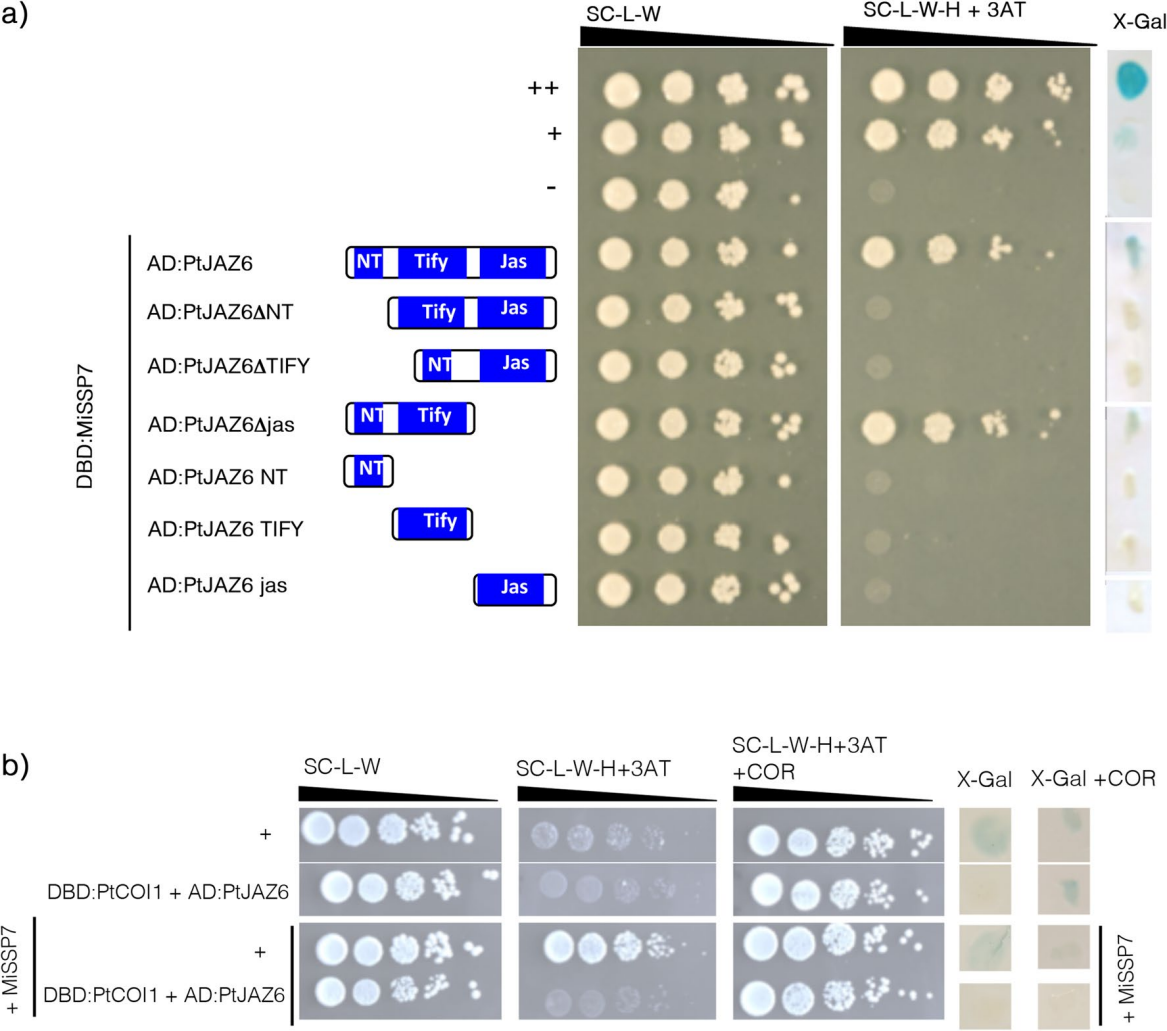
Both NT and TIFY domains of PtJAZ6 are necessary for its interaction with MiSSP7. (**a**) Yeast-two hybrid (Y2H) assays to assess which domains of PtJAZ6 are required for its interaction with MiSSP7. MiSSP7 was fused to the GAL4 DBD, whereas full length or mutated versions of PtJAZ6 were fused with the GAL4 AD. (**b**) Yeast-three hybrid assay to assess if the presence of MiSSP7 impacts the interaction between PtJAZ6 and PtJAZ6-PtCOI1. PtJAZ6 was fused to the GAL4 AD and PtCOI1 was fused to GAL4 DBD. Coronatine (COR) triggers the interaction betwen PtJAZ6 and PtCOI1^12^. *TRP1*, *LEU2* and *URA3* served as transformation markers (the latter in case of triple hybrids) for vectors whereas *HIS3* and β-galactosidase LacZ (X-Gal) were used as reporter genes. 3-Amino-1, 2, 4-triazole (3-AT) was used to suppress self-activation of the HIS3 gene. The control colonies are shown in the upper part of the panel: ++ is a strong positive interaction, + is a weakly interacting pair,— is a negative control (no interaction). 3-Amino-1, 2, 4-triazole (3-AT) was used to suppress self-activation at the *HIS3* gene. White lines indicate cropped and repositioned images while the full-length blots and gels are presented in Supplementary Information online.

We previously demonstrated that the fungal effector MiSSP7 from the ECM basidiomycete *Laccaria bicolor* binds to PtJAZ6 and alters its stability^12^. To determine which domain of PtJAZ6 is required for the interaction with MiSSP7, we co-expressed MiSSP7 and the different variants of PtJAZ6 in yeast cells. Yeast growth and β-galactosidase activity assays showed that MiSSP7 could only interact with the full length JAZ6 and the Jas domain-deleted version of PtJAZ6 but not with any other deleted version (Fig. 4 and Supplementary Figure S4b). From these data we conclude that both NT and TIFY domains are required for the interaction of PtJAZ6 with MiSSP7. In a previous study, we showed that PtJAZ6 interacts with PtCOI1 in a JA (coronatine)-dependent fashion^12^. Therefore, to test whether MiSSP7 hinders the binding of PtCOI1 to PtJAZ6, we used a yeast-three-hybrid (Y3H) assay. Yeasts co-expressing AD:PtJAZ6 and DBD:PtCOI1 were transformed with the *MiSSP7* gene. We tested the expression of the two reporter genes *HIS3* and *lac*Z in these triple-hybrid strains, in the presence of coronatine to trigger the interaction between PtJAZ6 and PtCOI1. The growth of double- and triple-hybrid clones was similar on the selective medium without histidine, whereas the triple-hybrid clones displayed no detectable β-galactosidase activity in the presence of coronatine (Fig. 4 and Supplementary Figure S4b). These results confirm the previous finding that MiSSP7 weakens but does not disrupt the PtJAZ6-PtCOI1 interaction^11^.

### MiSSP7 affects PtJAZ6 homo- and heterodimerization

**Figure 5:**
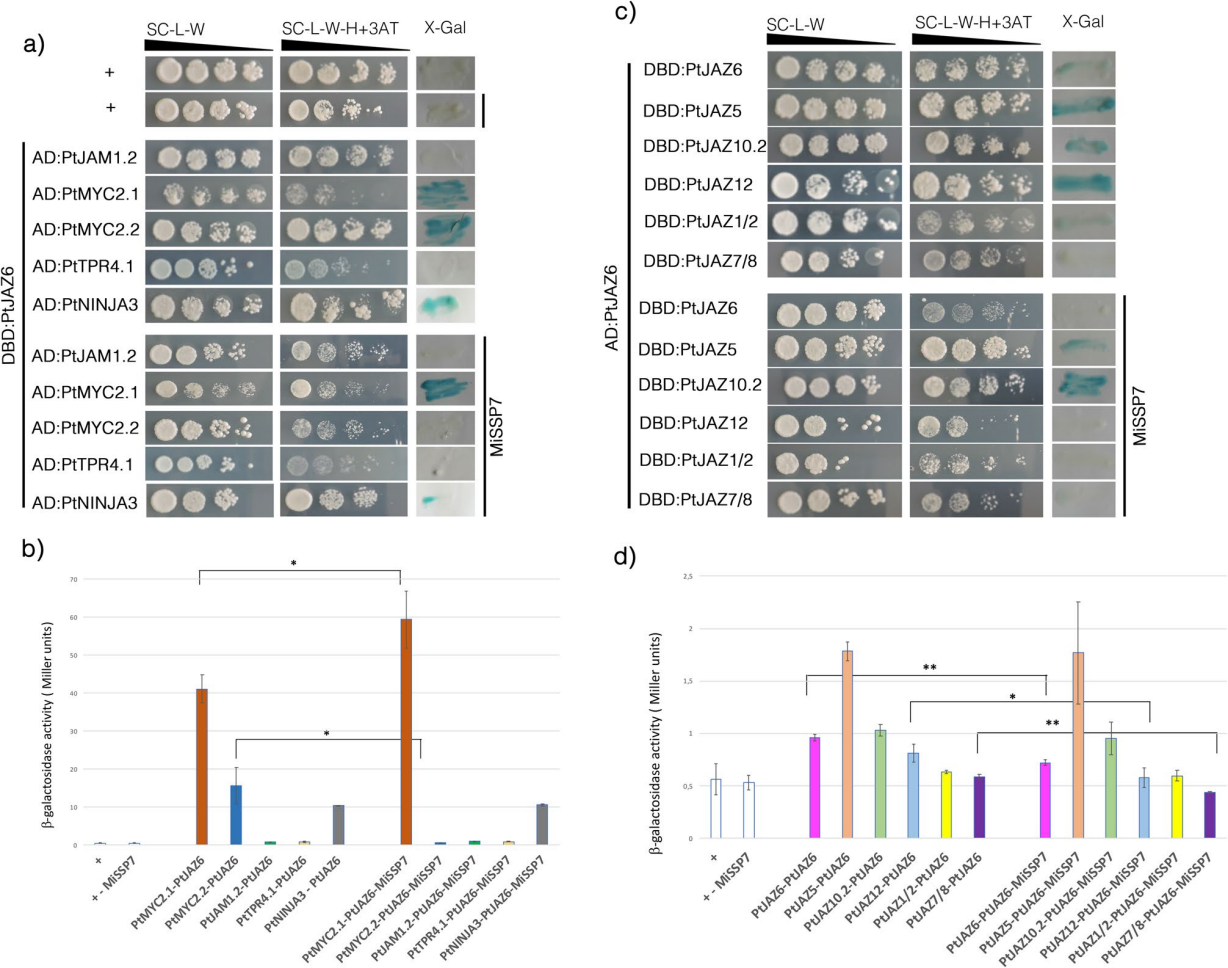
MiSSP7 impacts the structure of the jasmonate perception complex. (**a**, **b**) MiSSP7 differentially impacts the interaction between PtJAZ6 and the two isoforms of PtMYC2 but does not alter PtJAZ6-PtJAM1.2 or PtJAZ6-PtTPR4.1 interactions. PtJAZ6 was fused to the GAL4 DBD. PtMYC2s, PtJAM1.2, PtNINJA3 and PtTPR4.1 were fused with the GAL4 AD. (**c**, **d**) MiSSP7 antagonizes dimerization between PtJAZ6 and other PtJAZ proteins. PtJAZ6 was fused to the GAL4 AD and PtJAZ proteins were fused with the GAL4 DBD. For a) and c), yeasts were transformed with *MiSSP7* to give triple hybrids. *TRP1*, *LEU2* and *URA3* served as transformation markers (the latter in case of triple hybrid colonies) while *HIS3* and β*-galactosidase LacZ* (X-Gal) were used as reporter genes. 3-Amino-1, 2, 4-triazole (3-AT) was used to suppress self-activation of the *HIS3* gene. The control colonies are shown in the upper part of the panel: + is a weakly interacting pair. For b) and d), the enzymatic activity of the β-galactosidase in different yeast colonies was quantified via ONPG assay. Significant differences between yeast colonies transformed or not with MiSSP7 were assessed via pairwise Student’s t-test (*p < 0.05, **p < 0.01). White lines indicate cropped and repositioned images while the full-length blots and gels are presented in Supplementary Information online.

To assess the role of the MiSSP7 protein in the modulation of the interaction between PtJAZ6 and its partner proteins we conducted a Y3H assay. Yeast strains expressing DBD:PtJAZ6 together with PtMYC2s, PtJAM1.2, PtTPR4.1, PtNINJA3 or other PtJAZ proteins fused to GAL4 AD were transformed with the *MiSSP7* gene and the expression of the reporter genes *HIS3* and *lac*Z was assessed. We were unable to recover triple hybrids expressing AD:PtNINJA1 and AD:PtJAM1.1 despite three tries; therefore, we could not test if MiSSP7 influenced the PtJAZ6-PtJAM1.1 or PtJAZ6-PtNINJA1 interaction. Nevertheless, yeast colonies expressing AD:PtMYC2.1, DBD:PtJAZ6 and MiSSP7 grew better on medium lacking histidine and showed an enhanced β-galactosidase activity assay with respect to yeasts expressing AD:PtMYC2.1 and DBD:PtJAZ6 only. An opposite result was observed in case of colonies expressing AD:PtMYC2.2, while no difference was seen in case of yeasts expressing AD:PtJAM1.2, AD:PtNINJA3 or AD:PtTPR4.1 (Fig. 5a,b). These results suggest that the presence of MiSSP7 appears to improves/strengthens the interaction between PtJAZ6 and PtMYC2.1 and impedes/weakens the interaction between PtJAZ6 and PtMYC2.2. Since MiSSP7 cannot bind MYC2.1 or PtMYC2.2 alone (Supplementary Figure S5a), this effect is not due to MiSSP7-mediated activation of these TFs. On the other hand, MiSSP7 did not impact the interaction between PtJAZ6 and PtJAM1.2, PtJAZ6 and PtNINJA3 or PtJAZ6 and PtTPR4.1 (Fig. 5a,b).

We also tested whether the expression of *MiSSP7* affected the formation of PtJAZ6 homo- and heterodimers. We detected diminished β-galactosidase activity upon MiSSP7 expression in yeasts harboring DBD:PtJAZ6, DBD:PtJAZ5, DBD:PtJAZ12 or DBD:PtJAZ1/2 together with AD:PtJAZ6. Moreover, yeasts expressing AD:PtJAZ6, DBD:PtJAZ6 and MiSSP7 displayed reduced growth on selective medium lacking histidine with respect to AD:PtJAZ6/DBD:PtJAZ6-harboring yeasts (Fig. 5c,d). However, MiSSP7 did not promote PtJAZ6 interaction with non-interacting PtJAZ proteins (Supplementary Figure S5b). These results suggest that MiSSP7 specifically antagonizes the formation of PtJAZ6-PtJAZ6 homodimers and of PtJAZ6-PtJAZ5, PtJAZ6-PtJAZ12 and PtJAZ6-PtJAZ7/8 heterodimers.

## Discussion

Comparative genomic and phylogenetic analyses of genes encoding the core components of the JA-Ile signaling pathway have highlighted its conservation throughout evolution from the last common ancestor of land plants^52^. However, the presence of orthologs of JA signaling components in a plant genome does not determine the composition of the JA-signaling complex in a precise biological context and environment. Furthermore, studies of plant immunity demonstrated that plant-associated microorganisms manipulate JA-signaling through the use of effectors; some interfere with JA-Ile biosynthesis^30,46,47,49^, others modify the stability of JAZ repressors^12,41–43^, while yet others hamper the activity of JA-regulated TFs^44,45^. The molecular mechanisms behind these outcomes vary considerably. For instance, *Pseudomonas syringae* effectors HopZ1 and HopX1 promote JAZ degradation, thus activating JA-dependent responses to antagonize SA-induced responses and promoting bacterial infection. These effectors achieve this through distinct molecular mechanisms: HopX1 is a cysteine protease cleaving the central ZIM domain of JAZ proteins^41^ whereas HopZ1 acetylates soybean and *Arabidopsis* JAZ proteins^42^. Recent studies demonstrated that effectors can also target TFs mediating responses to JA. For example, the tomato yellow leaf curl China virus βC1 effector interacts with MYC2 to inhibit the expression of terpene synthase genes^45^. The *P. syringae* HopBB1effector also has a dual function, as it dissociates the *Arabidopsis* JAZ3-MYC2 complex and connects JAZ3 to the TCP14 TF, leading to TCP14 degradation^44^. Therefore, the HopBB1 effector modulates two distinct JA-dependent transcriptional modules. Finally, MiSSP7 from the mutualistic fungus *L. bicolor* prevents PtJAZ6 degradation^12^ but the molecular mechanism underpinning this phenomenon has not been yet deciphered.

**Figure 6:**
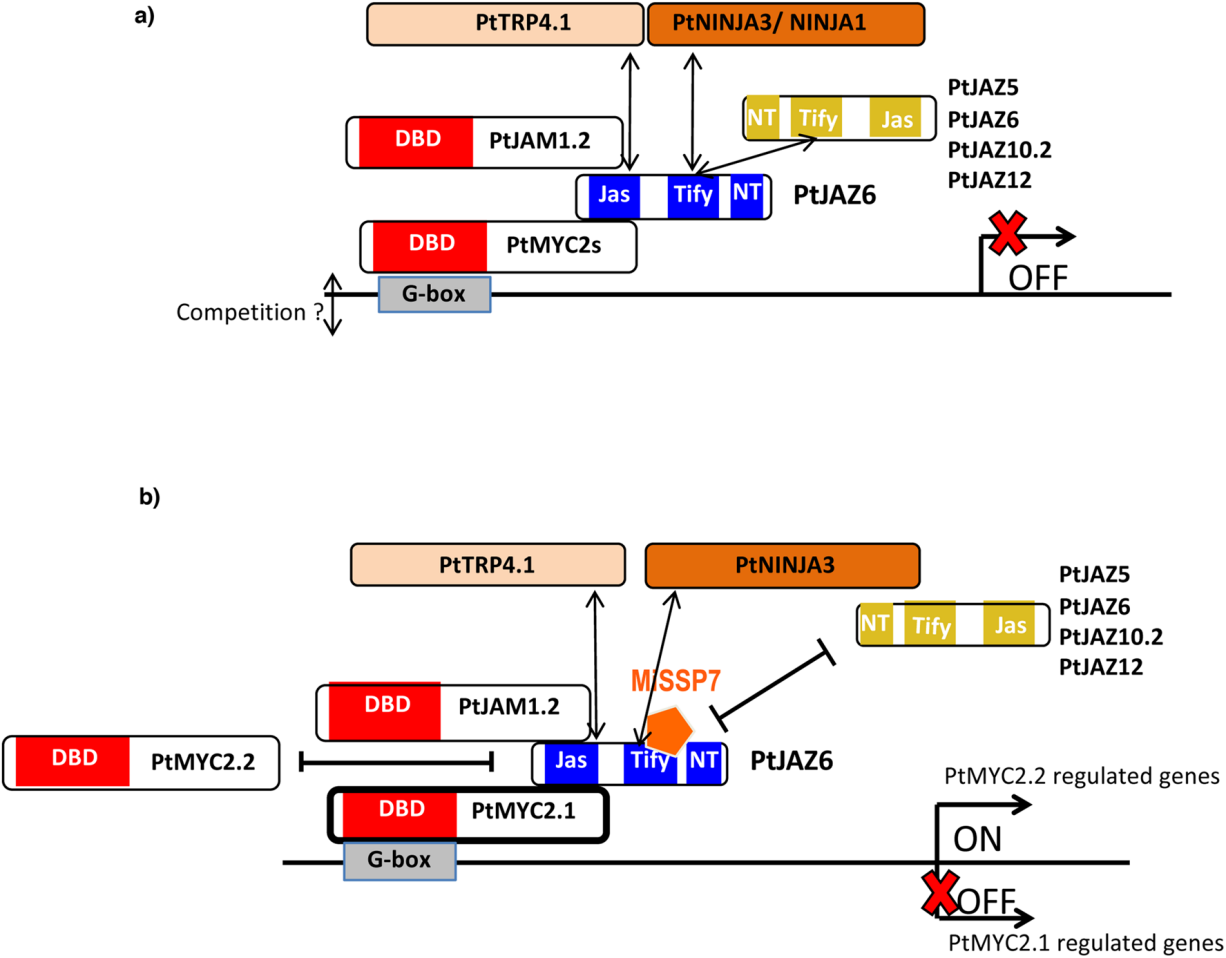
PtJAZ6-interacting proteins in *Populus* ectomycorrhizal root tips. (**a**) PtJAZ6 physically interacts with the transcription factors PtMYC2s and PtJAM1s, as well as with PtTPR4.1, NINJA1 and NINJA3. It also forms homodimers and heterodimers with PtJAZ5, PtJAZ10.2 and PtJAZ12. PtJAZ6 probably suppresses the transcriptional activity of PtMYC2s and PtJAM1s by recruiting the general repressor PtTPR4.1 to the promoter of MYC2-responsive genes. (**b**) The fungal symbiotic effector MiSSP7 interacts with PtJAZ6^12^ inhibiting homo- and heterodimerization of PtJAZ6. Such inhibition may alter the stability of PtJAZ6. The presence of MiSSP7 does not impair the interaction between PtJAZ6 and PtNINJA1 or PtNINJA3 However, MiSSP7 loosens the interaction between PtJAZ6 and PtMYC2.2, while strengthening the interaction between PtJAZ6 and PtMYC2.1. MiSSP7-mediated enhancement of the PtJAZ6-PtMYC2.1 interaction probably dampens the transcriptional activity of PtMYC2.1. On the other hand, the release of PtMYC2.2 from PtJAZ6-mediated repression may activate gene expression depending on this transcription factor.

In this study, we identified the poplar proteins interacting with the JA-signaling repressor PtJAZ6. Further, we studied how these protein–protein interactions might be affected in ECM poplar roots by studying the impact of the *L. bicolor* effector MiSSP7 on the interactions between PtJAZ6 and its associated proteins (Fig. 6). We reasoned that MiSSP7 might dampen or enhance the strength of the interaction between PtJAZ6 and other poplar proteins through competition or bridging, respectively. We found that, similar to *Arabidopsis*, PtJAZ6 interacts with PtMYC2 and PtJAM1 transcription factors, as well as with one TOPLESS-related protein, PtTPR4.1, two NINJA proteins, PtNINJA1 and PtNINJA3, and other PtJAZ proteins (Fig. 6a). In addition, the presence of MiSSP7 qualitatively enhanced the interaction between PtJAZ6 and PtMYC2.1, while negatively impacting the interactions between PtJAZ6 and PtMYC2.2 or several PtJAZ proteins (Fig. 6b).

Furthermore, we demonstrated that the interaction between PtJAZ6 and PtMYC2.1, PtMYC2.2 or PtJAM1.1 requires the PtJAZ6 Jas domain, similar to *Arabidopsis*. AtJAMs are transcriptional repressors whereas AtMYC2 is a transcriptional activator. These proteins compete for binding to the G-box in the promoter of target genes^35^ and the activity of both is inhibited by AtJAZ proteins^17,27,36^. Since the DBDs of PtMYC2s and PtJAM1s are highly similar (Supplementary Figure S2a,b), we can speculate that PtMYC2s and PtJAM1s directly compete for DNA-binding sites. We also hypothesize that these transcription factors compete for binding to PtJAZ6, leading to either attenuation of JA-signaling or release of repression. It will be interesting to investigate whether cell-specific or mycorrhizal stage-specific modulation of PtMYC2s and PtJAM1s activity contributes to the attenuation of JA-signaling during mutualistic tree-microbe interactions. The expression of *PtMYC2s* and *PtJAM1s* was unaltered during the development of ectomycorrhiza *in vitro*^53,54^. However, post-translational modifications, adapted protein turnover rates or enhanced interactions with repressors proteins like PtJAZ6 may regulate DNA binding and activity of these transcription factors^55–59^. For example, our data suggested that MiSSP7 induces preferential binding of PtJAZ6 to PtMYC2.1 rather than to its paralogous protein PtMYC2.2. These results suggest neo-functionalization of the two paralogues PtMYC2.1 and PtMYC2.2 in poplar roots and hint at differential regulation of distinct branches of JA-signaling upon ECM establishment, with preferential repression of the PtMYC2.1-regulated branch (Fig. 6b). We previously demonstrated that inoculation of poplar roots with *L. bicolor* reduces the expression of some JA-marker genes^12^ and dampens root sensitivity to exogenous JA^54^. On the other hand, moderate activation of JA-signaling may contribute to ECM development^54^. Therefore, specific activation or dampening of JA-responsive genes during ectomycorrhiza formation may rely on differential regulation of PtMYC2.1 and PtMYC2.2 activity. Identifying genes specifically regulated by PtMYC2.1 or PtMYC2.2 in ectomycorrhizae will allow testing this hypothesis.

Based on the results of Y2H assays, we also demonstrated that PtJAZ6 interacts directly with one poplar TOPLESS-related protein through its EAR motif and with two NINJA proteins through its TIFY domain (Fig. 3c,d), similar to the *Arabidopsis* AtJAZ6^60^ and to four additional AtJAZ proteins^50,51,59^. In addition, PtJAZ6 interacts with itself and other PtJAZs proteins. The strongest interactions occurred within the homodimer PtJAZ6-PtJAZ6 and the heterodimers PtJAZ6-PtJAZ5, PtJAZ6-PtJAZ10.2 and PtJAZ6-PtJAZ12. Contrasting reports have been published on the interactome of AtJAZ6, ranging from no dimerization to weak dimerization with AtJAZ5 and AtJAZ6, medium-strength dimerization with JAZ10.1 and JAZ12 and strong dimerization with JAZ2^32,33^. Interestingly, we found that the interaction of MiSSP7 with PtJAZ6 antagonized its homo- and heterodimerization. Whether this dynamic has a stabilizing effect on the repressor protein PtJAZ6 remains to be investigated, since the in vivo function of JAZ dimerization is unknown. However, since protein dimerization is a common regulatory mechanism in signal transduction^61^, we could speculate that different PtJAZ dimers might generate diverse JA-signaling outputs. Alternatively, the formation of PtJAZ complexes with a specific range of stability may modulate the amplitude and the duration of JA-dependent responses^33,55^.

In a previous study, we showed that the *L. bicolor* symbiotic effector MiSSP7 prevents PtJAZ6 degradation in presence of JAs. Here, we provide evidence that this mutualistic effector modifies the dynamics of PtJAZ6 protein–protein interaction networks. More precisely, upon interaction with PtJAZ6, this effector may maintain the repression over PtMYC2.1-regulated genes, one of the JA-dependent transcriptional modules of poplar roots. It remains to be understood how such changes are required for successful root colonization by *L. bicolor*. Moreover, it remains to be demonstrated whether this mechanism is conserved for other species of ECM fungi.

## Materials and methods

### Gene cloning and editing of PtJAZ6

*PtJAZs*, *PtMYC2s*, *PtJAM1s*, *PtTPLs*, *PtNINJAs*, and *PtCOI1* as well as full-length and domain-deleted versions of *PtJAZ6,* were PCR-amplified from *Populus trichocarpa* root cDNA and cloned into entry (pDONR222/pDONR207) and destination vectors (pDEST22/ pDEST32/ pB7WGF2.0 or pK7WGR2.0) using Gateway recombination (ThermoFisher Scientific, Germany). Point mutations in *PtJAZ6* were generated with the QuickChange II XL Site-Directed Mutagenesis Kit (Agilent, Germany) on the entry vector pDONR222:PtJAZ6. A list of the primers used in this study is available in Table S2. The *MiSSP7* gene (Lacbi2 protein ID: 298,595) lacking its N-terminal signal peptide was PCR-amplified from cDNA synthetized using total RNA extracted from *L. bicolor-*colonized *P. trichocarpa* roots as template. This gene was then cloned into entry (pDONR222) and destination (pZMU-Dest^62^) vectors. The latter vector bears the *URA3* gene as auxotrophic genetic marker.

### Yeast two-hybrid screen

A yeast two-hybrid (Y2H) screen with PtJAZ6 as bait and Y2H assays were carried out as previously described^63,64^, using a cDNA library constructed from *L. bicolor-Populus* ECM root tips. *Populus* gene and protein IDs used in this study are listed in Table S1. MaV203 (*MATα; leu2-3,112; trp1-901; his3Δ200; ade2-101; gal4Δ; gal80Δ; SPAL10*_*UASGAL1*_*::URA3; GAL1::lacZ; HIS3*_*UASGAL1*_*::HIS3@LYS2; can1*^*R*^*; cyh2*^*R*^) and MaV103 (*MATa; leu2-3,112; trp1-901; his3Δ200; ade2-101; gal4Δ; gal80Δ; SPAL10 *_*UASGAL1*_*::URA3; GAL1::lacZ; HIS3*_*UASGAL1*_*::HIS3@LYS2; can1R; cyh2R*) yeast strains were used. Three control yeasts colonies are included: Krev1/RalGDS-*wt* as a strong positive interaction, Krev1/RalGDS-*m1* as a weak interaction, and Krev1/RalGDS-*m2* as a no-interaction control (ProQuest™ Ywo-Hybrid system, ThermoFisher Scientific). The three reporter genes available within the system are *URA3*, *HIS3*, and *LacZ*, allowing yeast growth on medium lacking uracil or histidine, or the production of the β-galactosidase enzyme, respectively. Amino-1, 2, 4-triazole (3-AT) at 25 mM was used to suppress self-activation of the *HIS3* gene.

### Yeast triple hybrid assays

MaV203 yeast containing the vectors pDEST22 and pDEST32 with various constructs were grown in 5 mL of liquid SC-LW medium in shaker at 28 °C and 200 rpm for 16 h. Cultures were then diluted to an OD_600_ of 0.6 in a 5 mL volume of liquid SC-L-W medium and grown in a shaker using the same conditions until they reached an OD_600_ of 2. Cultures were subsequently centrifuged for 5 min at 4 °C and 3000 rpm for five minutes and washed three times with sterile water. Twenty-nine μL of the washed yeast culture were added to the transformation mix (240 μL of 50% PEG3350, 36 μL of 1 M LiAC, and 50 μL of 2 mg/mL salmon sperm ssDNA) and in presence of 200 ng of pZMU-Dest plasmid in which *MiSSP7* was cloned. Tubes were mixed, then incubated in a water bath at 42 °C for 45 min. Tubes were then centrifuged for 5 min at 9000 rpm and the supernatant discarded. The pellet was resuspended in 50 μL of liquid SC-L-W medium and plated on SD-L-W-U plates. Plates were sealed with parafilm and incubated in static oven at 28 °C for 72 h. Transformed colonies were tested via PCR for presence of *MiSSP7* and then stored in glycerol in freezer at − 80 °C. The Drop test was similarly performed for Y3H assays as for the Y2H assays, with the difference that all solid and liquid media also lacked uracil, to select for the *MiSSP7*- containing vector pZMU-Dest.

### Qualitative β-galactosidase reporter assay on filters

The activity of the yeast β-galactosidase, a subunit which is coded by the *GAL*1::*lac*Z reporter, was tested according to a previously published method^64^. Briefly, yeast patches were replica-plated onto a YAPD plates covered by 0.45 μM nitrocellulose filters (Hybond-C, Amersham,GE-Healthcare, USA) and incubated overnight in static incubator at 30 °C. Two Whatman filter papers were placed in an empty 15-cm Petri dish and soaked with 3 mL of Z-buffer X-Gal solution (16.1 g/L Na_2_HPO_4_*7H_2_O, 5.5 g/L NaH_2_PO_4_*H_2_O, 0.75 g/L KCl, 0.246 g/L MgSO_4_*7H_2_O, pH 7.0) supplemented with 1.83 μL/mL of β-mercaptoethanol and 16.66 μL/mL of 4% X-Gal (4-Methylumbelliferyl-beta-D-galactopyranoside, BiosynthCarbosynth, Switzerland). The nitrocellulose membranes with the yeast streaks were then detached from the YAPD plates, submitted to two freeze–thaw cycles in liquid nitrogen and placed on the Z-buffer-soaked Whatman filter papers. Plates were incubated at 37 °C for ~ 6 h, after which pictures were taken. The white color indicates lack of β-galactosidase activity and thus lack of expression of the reporter construct *GAL*1::*lac*Z, while progressively darker blue color indicates stronger β-galactosidase activity and thus stronger interaction between AD- and DBD-fused proteins.

### Quantitative β-galactosidase reporter assay

We quantified the enzymatic activity of the β-galactosidase using ONPG (ortho-Nitrophenyl-β-galactoside, ThermoFisher Scientific, Germany) as substrate according to the Yeast Protocols Handbook (Protocol No PT3024-1, Version No PR973283, Takara Bio, USA). Briefly, ONPG was dissolved at 4 mg/mL in Z-buffer with shaking for 1–2 h. Two mL from 5-mL overnight yeast cultures in liquid SD selective medium were transferred onto fresh YPAD liquid medium and incubated at 28 °C for 5 h with shaking (250 rpm). At the end of incubation, the exact OD_600_ was recorded. Yeast cells were retrieved by centrifugation at 14,000 rpm for 30 s and washed once with 1.5 mL of Z-buffer, then resuspended in 300 μL of Z-buffer. A hundred μL of resuspended yeast cells were transferred to fresh microcentrifuge tubes and underwent three freeze–thaw cycles by being transferred between liquid nitrogen and a 37 °C water bath every minute. Seven hundred μL of Z-buffer supplemented with 0.27% v/v β-mercaptoethanol were added to each tube. Finally, after addition of 160 μL of Z-buffer supplemented with ONPG, the tubes were placed in a 30 °C static incubator for 16 h. The reaction was stopped through the addition of 400 μL of 1 M Na_2_CO_3_. Tubes were then centrifuged for 10 min at 14,000 rpm and the OD_420_ of the supernatants was read through a Tecan Infinite 200 PRO plate reader (TECAN, Austria). The enzymatic activity of the β-galactosidase for each yeast colony was expressed in Miller units, as follow: Miller units = 1000 × OD_420_/(t x V x OD_600_), where t corresponds to the elapsed time of incubation (min) and V = 0.1 mL x concentration factor.

### Plant material and growth conditions

*Nicotiana benthamiana* seeds were germinated and grown for three weeks in a climate chamber at 24 °C, under a 16 h photoperiod, with 70% relative humidity.

### Agroinfiltration of tobacco leaves and localization of the fusion proteins in epidermal cells

*Agrobacterium tumefaciens GV3101* transformed with pB7WGF2.0 or pK7WGR2.0 harboring each gene of interest or empty (as control) were cultivated overnight at 28 °C in Luria–Bertani medium supplemented with 10 μg/mL gentamycin, 50 μg/mL spectinomycin and 50 μg/mL rifampicin. After centrifugation, bacterial pellets were resuspended in infiltration buffer (10 mM MgCl_2_, 10 mM MES, 200 μM Acetosyringone, pH 5.6) and incubated for 2 h at 28 °C with gentle shaking. Bacterial cultures were then mixed at a 1:1 ratio to a final OD_600_ of 0.6 before infiltration of three-week-old tobacco leaves. In order to increase the expression of the transgene and counteract its potential silencing, an *A. tumefaciens* GV3101 strain expressing the tomato bushy virus (TBSV) suppressor of post-transcriptional gene silencing – P19 was added to the mix at a final OD_600_ of 0.1. Infiltration buffer was used as negative control. After a 72 h-incubation in a climate chamber (24 °C, 16 h photoperiod, 70% relative humidity), leaf discs were harvested, and mounted between slide and coverslip (glycerol 20%). Live-cell imaging was performed using 10 × (air) and 40 × (water immersion) objectives of the confocal microscope Zeiss LSM780 (Zeiss, Germany). The GFP was excited at 488 nm, whereas the mCherry was excited at 561 nm. Specific emission signals corresponding to the GFP and the mCherry were collected at 505–525 and 580–620 nm, respectively. Alternatively, leaves were harvested and flash-frozen for *in planta* co-immunoprecipitation.

### In planta co-immunoprecipitation

Three days after bacterial infiltration, four leaves per transformation were harvested and ground using liquid nitrogen. The resulting powder was resuspended in 10 mL of extraction buffer (10% glycerol, 25 mM Tris pH 7.5, 1 mM EDTA, 150 mM NaCl, 5 mM DTT, 0.1% Tergitol NP40S, 200 mg PVPP and 100 μL protease inhibitor [Stock 100x, Sigma-Aldrich, France]. PVPP was added in the buffer the night before the extraction while DTT, tergitol and protease inhibitors were added just prior to extraction. Samples were incubated for 10 min at 4 °C in a rotator and then centrifuged at 9000 rpm for 5 min at 4 °C. The supernatant was filtered using a cell strainer with 70 μm-wide pores. About 500 μL of supernatant was TCA-precipitated and pellet was resuspended with 60μL of Laemmli buffer + DTT (5 mM) and conserved on ice (total protein fraction or input). Magnetic beads coupled with anti-GFP antibodies (GFP-Trap_M, Chromo Tek, Germany) were used for CoIP. Before use, they were washed twice using washing buffer (10 mM Tris–HCl pH 7.5, 150 mM NaCl, 0,5 mM EDTA and protease inhibitors). Each supernatant was mixed with 30 μL of magnetic beads and incubated for 20 min at 4 °C in a rotator with slight agitation. Beads were then collected, washed five times with 1 mL of washing buffer and resuspended in 75 μL of Laemmli buffer 1x + DTT (5 mM) (coimmunoprecipitated fraction).

### Western blot

The input and coimmunoprecipitated fractions were incubated for 5 min at 95 °C and then centrifuged for 1 min at 9000 rpm. Aliquots of 15 μL were separated on polyacrylamide gels (Mini-Protean TGX Precast Gels 4–15%, Bio-Rad, France), followed by a transfer onto PVDF membranes Trans-Blot Turbo Transfer System, Bio-Rad, France). After transfer, membranes were stained with Ponceau red to assess total protein loading, then de-stained with 0.1 M NaOH and washed with distilled water. Membranes were blocked for 2 h at 4 °C and 50 rpm in blocking buffer (PBS 1x, 1% casein) followed by incubation with the anti-GFP (GFP Antibody (B-2) HRP, Santa Cruz Biotechnology, USA, 200 μg/mL) or anti-RFP (RFP Tag Antibody (RF5R), ThermoFisher Scientific, Germany, 1 mg/mL) primary antibody diluted 1/1000 in blocking buffer overnight at 4 °C and 50 rpm. The anti-GFP antibody is conjugated to HRP allowing a direct detection on the membranes. Membranes incubated with the anti-RFP primary antibody were washed four times for 5 min with PBS + 0.05% Tween 20 and incubated with a secondary antibody conjugated to HRP (ab6789, Abcam, United-Kingdom, 2 mg/mL) diluted 1/10,000. After immunolabelling, membranes were washed four times for five minutes with PBS + 0.05% Tween 20 and imaged 5–7 min after addition of five mL of substrate (Clarity Western ECL Substrate, Bio-Rad, France). Pictures were taken using the ChemiDoc XRS + Imaging System (Bio-Rad, France).

### Phylogenetic analysis

Evolutionary analyses were conducted in MEGA7^65^. In all cases, the evolutionary history was inferred by using the Maximum Likelihood method based on the JTT matrix-based model^66^. The tree with the highest log likelihood is shown. Initial trees for the heuristic search were obtained automatically by applying Neighbor-Joining and BioNJ algorithms to a matrix of pairwise distances estimated using a JTT model, and then by selecting the topology with superior log likelihood value. The trees were drawn to scale, with branch lengths measured in the number of substitutions per site. Five hundred bootstraps were performed to assess the robustness of the data. FigTree (https://tree.bio.ed.ac.uk/software/figtree/) was used to visualize and annotate the tree.

## Supplementary information

Supplementary Table S1.

Supplementary Table S2.

Supplementary Legends.
