# Supplementary material for: The mutualism effector MiSSP7 of Laccaria bicolor alters the interactions between the poplar JAZ6 protein and its associated proteins
Source: Sci Rep. 2020 Nov 23;10:20362. doi: 10.1038/s41598-020-76832-6 (PMC7683724; doi:10.1038/s41598-020-76832-6)
Supplement: Supplementary file 3 — Supplementary Legends. [file 41598_2020_76832_MOESM3_ESM.pdf]

The mutualism effector MiSSP7 of *Laccaria bicolor* alters the interactions between the poplar JAZ6 protein and its associated proteins.

Yohann Daguerre, Veronica Basso, Sebastian Hartmann-Wittulski, Romain Schellenberger, Laura Meyer, Justine Bailly, Annegret Kohler, Jonathan M. Plett, Francis Martin, Claire Veneault-Fourrey.

Supplementary Figure S1. PtJAZ6 does not auto-activate the three reporter genes used in the yeast-two hybrid system. PtJAZ6 was fused to the DNA-binding domain (DBD) of the GAL4 yeast transcription factor. *LEU2* served as transformation marker for the vector. *HIS3*, *URA3*, and *LacZ* (X-Gal) were used as reporter genes. 3-Amino-1, 2, 4-triazole (3-AT) was used to suppress self-activation of the *HIS3* gene. White lines indicate cropped and repositioned images.

Supplementary Figure S2. Features of *Populus* MYC2 and JAM1 proteins.
(a-b) Alignment of amino acid sequences of *Populus* and *Arabidopsis* JAM1 (a) and MYC2s (b) proteins. Alignments were performed using T-Coffee (http://tcoffee.crg.cat/apps/tcoffee/do:regular) and Boxshade (https://embnet.vital-it.ch/software/BOX_form.html) for publication-quality output. The DNA-binding domain and the bHLH-MYC N-terminal domain are indicated in blue and red, respectively.
(c) Autoactivation tests in yeast using PtMYC2.1, PtMYC2.2, or PtJAM1.1 fused to the DNA-binding domain of the GAL4-- yeast transcription factor. *LEU2* served as transformation marker for the vector while *HIS3* and *LacZ* (X-Gal) were used as reporter genes. 3-Amino-1, 2, 4-triazole (3-AT) was used to suppress self-activation of the *HIS3* gene. White lines indicate cropped and repositioned images.

Supplementary Figure S3. PtJAZ6 contains three typical domains of JAZ proteins.
The variable NT domain (blue) is required for the interaction with several distinct signal transductions factors such as DELLA proteins or HDA6. The TIFY (ZIM) domain (red) in the middle is required for the dimerization of JAZs. For most *Arabidopisis* JAZ proteins, it is also required to recruit TPL or TPRL proteins indirectly through the EAR motif contained in NINJA proteins. The C-terminal Jas domain (green) is highly conserved, contains the degron, and is required for binding to COI1 and TFs. The amino acid sequences of PtJAZ6 and AtJAZ6 were aligned using T-Coffee (http://tcoffee.crg.cat/apps/tcoffee/do:regular) and the alignment was formatted with Boxshade (https://embnet.vital-it.ch/software/BOX_form.html).

The EAR (Ethylene-responsive element binding factor-associated amphiphilic repression) motif is underlined.

Supplementary Figure S4. Quantification of the β-galactosidase activity by ONPG assay in the yeast colonies showed in Figure 1 and Figure 3 (a) and Figure 4 (b). Significant differences were assessed via pairwise Student's t-test (* = p<0.05, ** = p<0.01)

Supplementary Figure S5.
(a) MiSSP7 does not interact with either PtMYC2.1 or PtMYC2.2. PtMYC2.1 and PtMYC2.2 were fused to the GAL4 activation domain (AD) and MiSSP7 was fused to the GAL4 DNA binding domain (DBD). *LEU2* and *TRP1* served as transformation markers. *HIS3*, *URA3*, and *LacZ* (X-Gal) were used as reporter genes.
(b) MiSSP7 does not affect the weak interactions between PtJAZ6 and other PtJAZ proteins. PtJAZ6 was fused to with the GAL4 activation domain (AD). Other PtJAZ proteins were fused to GAL4 DNA-binding domain (DBD). Yeasts were transformed with *MiSSP7* to give triple hybrids. *TRP1*, *LEU2*, and *URA3* served as transformation markers while *HIS3* and *LacZ* (X-Gal) were used as reporter genes. 3-Amino-1, 2, 4-triazole (3-AT) was used to suppress self-activation of the *HIS3* gene. White lines indicate cropped and repositioned images while the full-length blots and gels are presented in Supplementary Information online.
(c) Quantification of β-galactosidase activity in yeast colonies showed in (b) by ONPG assay. + is a weakly interacting pair. Significant differences in interaction strength between PtJAZ proteins and the different deleted versions of PtJAZ6 in presence or absence of MiSSP7 were assessed via pairwise Student's t-test (* = p < 0.05, ** = p < 0.01).

Table S1. Expression level of poplar proteins involved in JA-signaling pathway in *in vitro* grown poplar-*L. bicolor* ectomycorrhiza and in poplar root tips. Data were extracted from the NCBI Bioproject PRJNA444231, PRJNA444232, PRJNA444233, PRJNA443960, PRJNA443961, PRJNA443962, PRJNA444765, PRJNA444766, PRJNA444185, PRJNA444207, PRJNA444208, PRJNA444209.

Table S2. List of primers used in the study

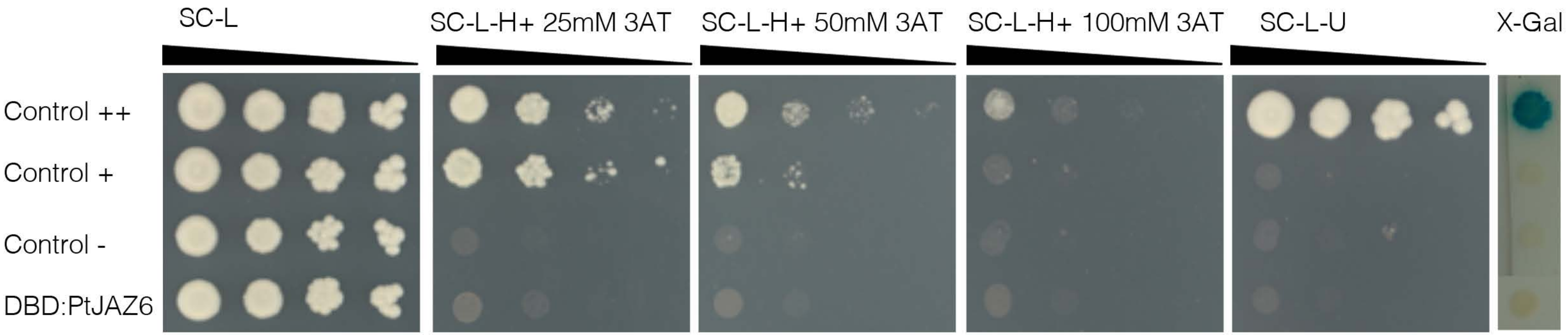
SC-L
SC-L-H+ 25mM 3AT
SC-L-H+ 50mM 3AT
SC-L-H+ 100mM 3AT
SC-L-U
X-Gal
Control ++
Control +
Control -
DBD:PtJAZ6

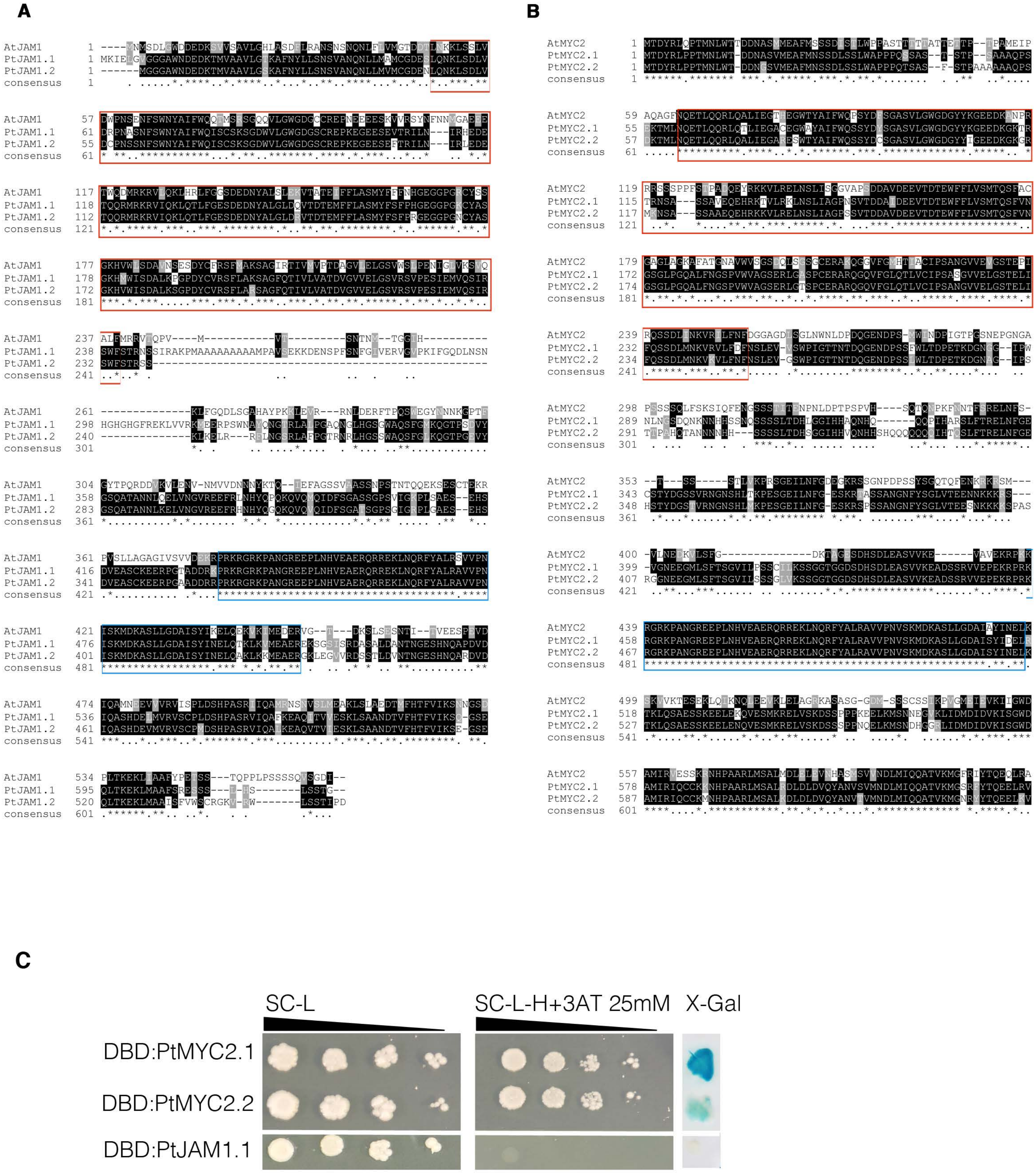
A
AtJAM1
PtJAM1.1
PtJAM1.2
consensus
B
AtMYC2
PtMYC2.1
PtMYC2.2
consensus
C
SC-L
SC-L-H+3AT 25mM
X-Gal
DBD:PtMYC2.1
DBD:PtMYC2.2
DBD:PtJAM1.1

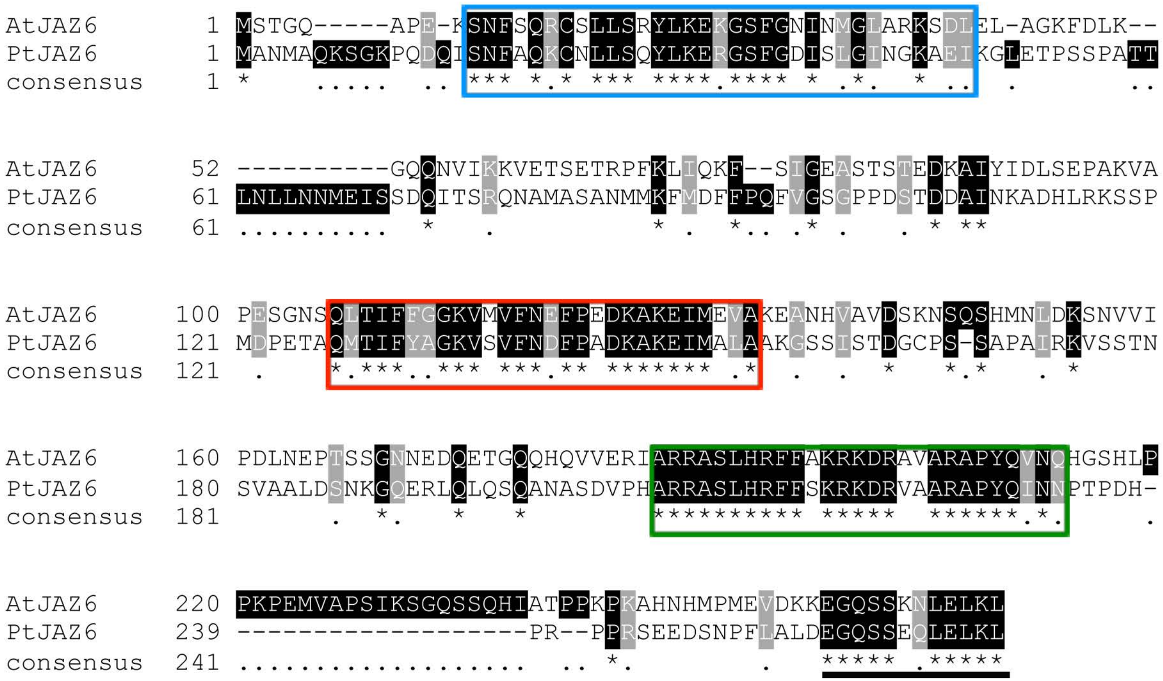
AtJAZ6 1 MSTGQ-----APE-KSNFSQRCSLLSRYLKEKGSFGNINMGLARKSDLEL-AGKFDLK--
PtJAZ6 1 MANMAQKSGKPQDQISNFAQKCNLLSQYLKERGSFGDISLGINGKAEIKGLETPSSPATT
consensus 1
AtJAZ6 52 ----------GQQNVIKKVETSETRPFKLIQKF--SIGEASTSTEDKAIYIDLSEPAKVA
PtJAZ6 61 LNLLNNMEISSDQITSRQNAMASANMMKFMDFFPQFVGSGPPDSTDDAINKADHLRKSSP
consensus 61
AtJAZ6 100 PESGNSQLTIFFGGKVMVFNEFPEDKAKEIMEVAKEANHVAVDSKNSQSHMNLDKSNVVI
PtJAZ6 121 MDPETAQMTIFYAGKVSVFNDFPADKAKEIMALAAKGSSISTDGCPS-SAPAIRKVSSTN
consensus 121
AtJAZ6 160 PDLNEPTSSGNNEDQETGQQHQVVERIARRASLHRFFAKRKDRAVARAPYQVNQHGSHLP
PtJAZ6 180 SVAALDSNKGQERLQLQSQANASDVPHARRASLHRFFSKRKDRVAARAPYQINNPTPDH-
consensus 181
AtJAZ6 220 PKPEMVAPSIKSGQSSQHIATPPKPKAHNHMPMEVDKKEGQSSKNLELKL
PtJAZ6 239 -------------------PR--PPRSEEDSNPFLALDEGQSSEQLELKL
consensus 241

a)

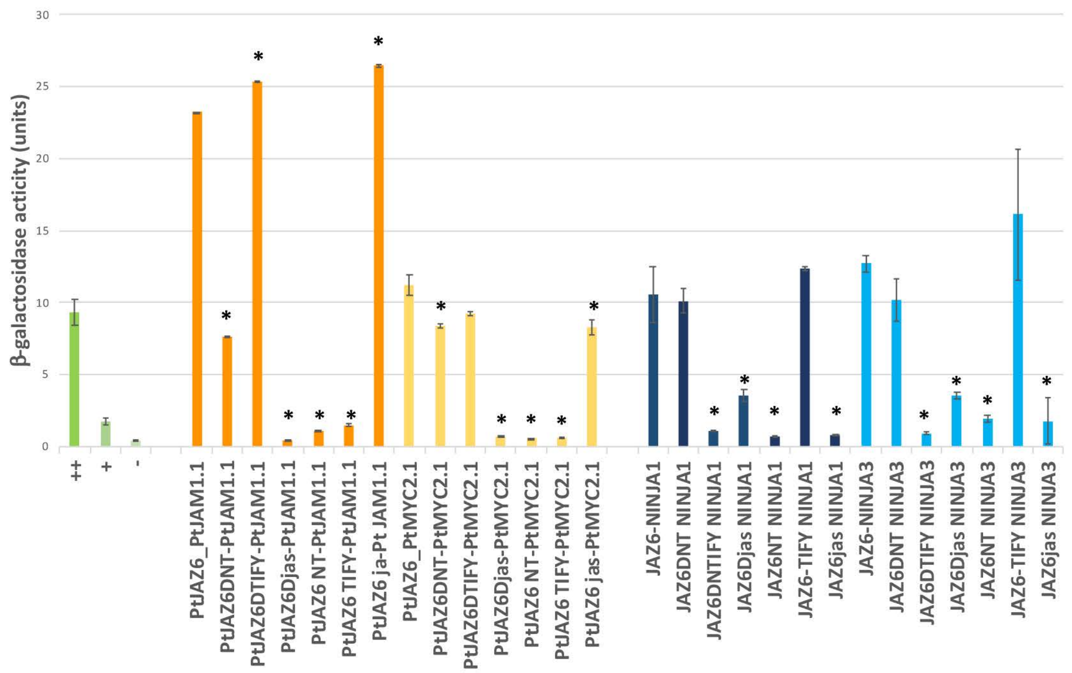
β-galactosidase acticity (units)
30
25
20
15
10
5
0
++
+
-
PtJAZ6_PtJAM1.1
PtJAZ6DNT-PtJAM1.1
PtJAZ6DTIFY-PtJAM1.1
PtJAZ6Djas-PtJAM1.1
PtJAZ6 NT-PtJAM1.1
PtJAZ6 TIFY-PtJAM1.1
PtJAZ6 ja-Pt JAM1.1
PtJAZ6_PtMYC2.1
PtJAZ6DNT-PtMYC2.1
PtJAZ6DTIFY-PtMYC2.1
PtJAZ6Djas-PtMYC2.1
PtJAZ6 NT-PtMYC2.1
PtJAZ6 TIFY-PtMYC2.1
PtJAZ6 jas-PtMYC2.1
JAZ6-NINJA1
JAZ6DNT NINJA1
JAZ6DNTIFY NINJA1
JAZ6Djas NINJA1
JAZ6NT NINJA1
JAZ6-TIFY NINJA1
JAZ6jas NINJA1
JAZ6-NINJA3
JAZ6DNT NINJA3
JAZ6DTIFY NINJA3
JAZ6Djas NINJA3
JAZ6NT NINJA3
JAZ6-TIFY NINJA3
JAZ6jas NINJA3

b)

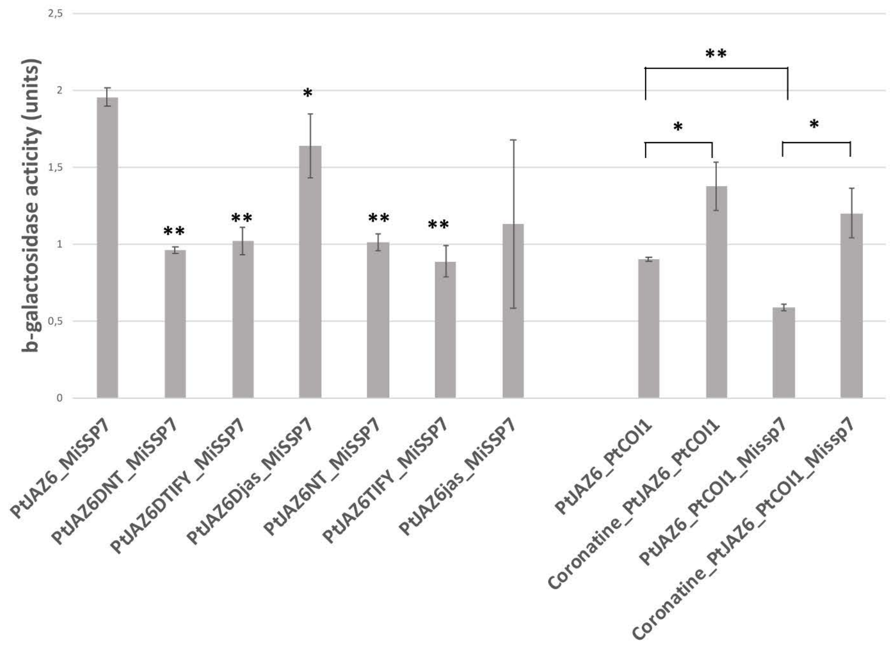
b-galactosidase acticity (units)
2,5
2
1,5
1
0,5
0
PtJAZ6_MiSSP7
PtJAZ6DNT_MiSSP7
PtJAZ6DTIFY_MiSSP7
PtJAZ6Djas_MiSSP7
PtJAZ6NT_MiSSP7
PtJAZ6TIFY_MiSSP7
PtJAZ6jas_MiSSP7
PtJAZ6_PtCOI1
Coronatine_PtJAZ6_PtCOI1
PtJAZ6_PtCOI1_Missp7
Coronatine_PtJAZ6_PtCOI1_Missp7

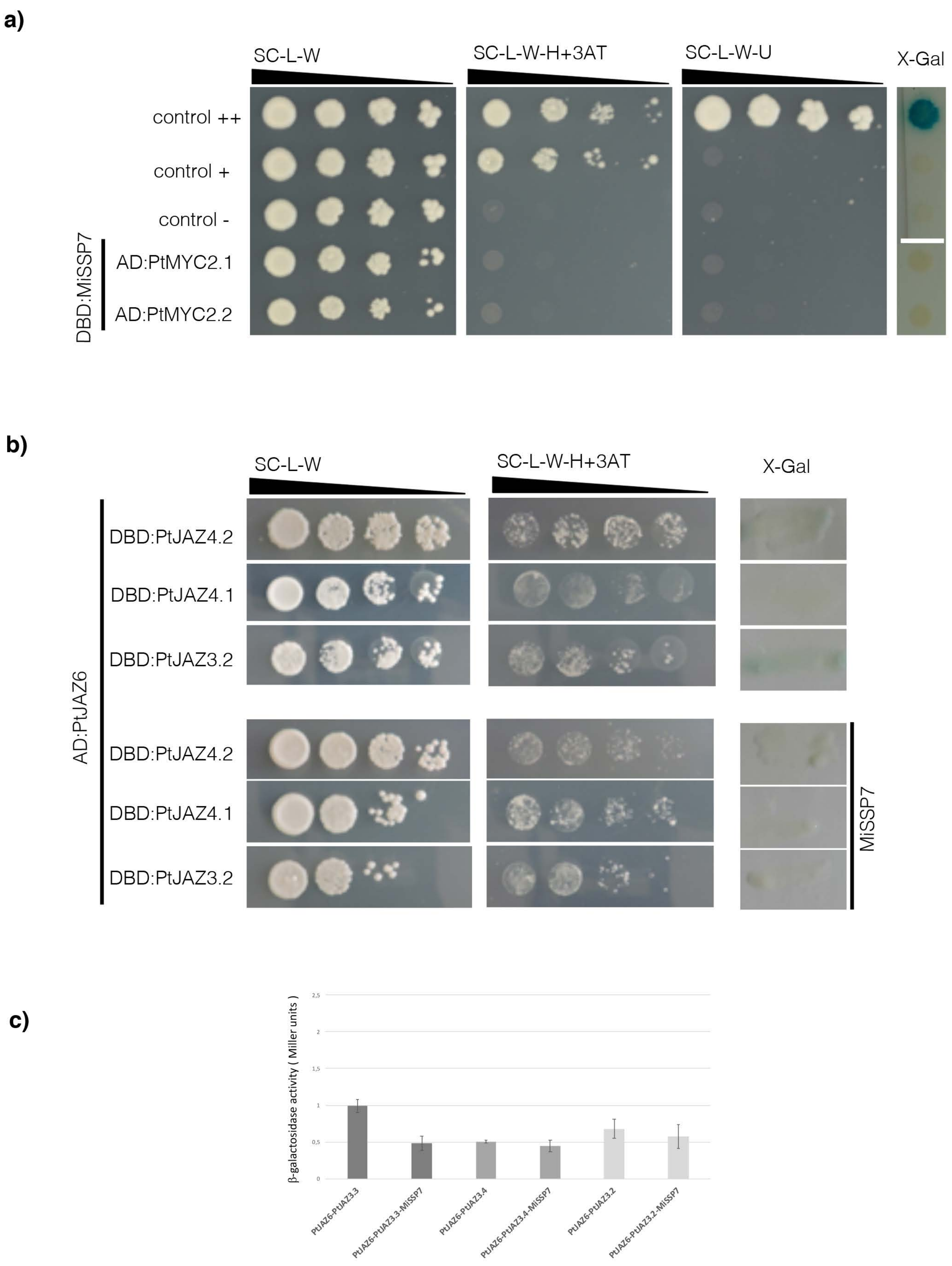
a)
SC-L-W
SC-L-W-H+3AT
SC-L-W-U
X-Gal
control ++
control +
control -
AD:PtMYC2.1
AD:PtMYC2.2
DBD:MiSSP7
b)
SC-L-W
SC-L-W-H+3AT
X-Gal
DBD:PtJAZ4.2
DBD:PtJAZ4.1
DBD:PtJAZ3.2
DBD:PtJAZ4.2
DBD:PtJAZ4.1
DBD:PtJAZ3.2
AD:PtJAZ6
MiSSP7
c)
β-galactosidase activity (Miller units)
2,5
2
1,5
1
0,5
0
PtJAZ6-PtJAZ3.3
PtJAZ6-PtJAZ3.3-MiSSP7
PtJAZ6-PtJAZ3.4
PtJAZ6-PtJAZ3.4-MiSSP7
PtJAZ6-PtJAZ3.2
PtJAZ6-PtJAZ3.2-MiSSP7

Supplementary information file.

The mutualism effector MiSSP7 of *Laccaria bicolor* alters the interactions between the poplar JAZ6 protein and its associated proteins *Yohann Daguerre, Veronica Basso, Sebastian Hartmann-Wittulski, Romain Schellenberger, Laura Meyer, Justine Bailly, Annegret Kohler, Jonathan M. Plett, Francis Martin, Claire Veneault-Fourrey.*

you will find almost all full blots or filters which have been cropped before being inserted in the figures.

Full original blots cropped for Figure 1 D. (PtJAM1.1 and PtJAZ6 co-immunoprecipitation)

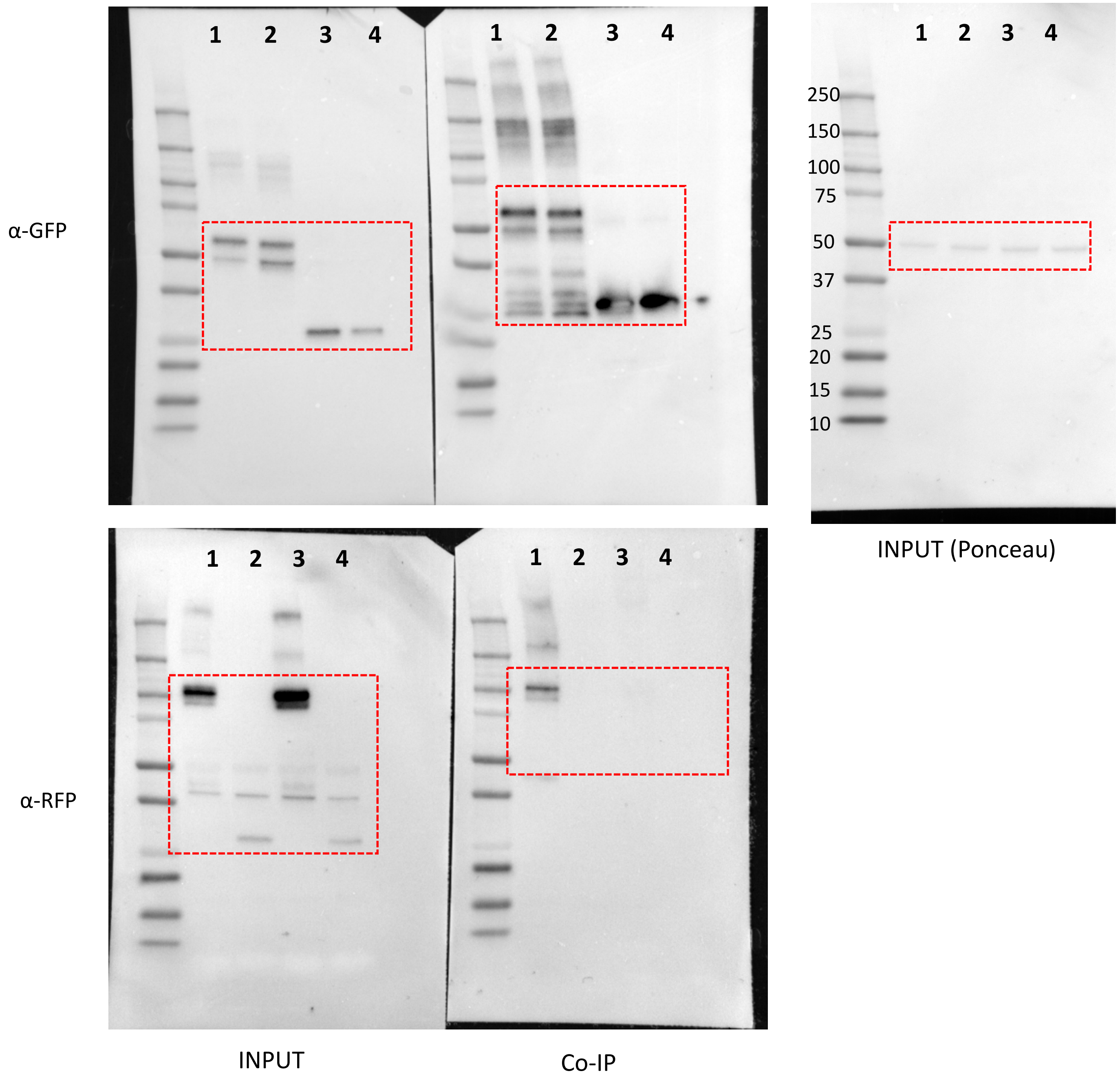

	1	2	3	4
PtJAZ6-GFP	+	+	-	-
PtJAM1.1-RFP	+	-	+	-
GFP	-	-	+	+
RFP	-	+	-	+

Full original blots cropped for Figure 1 D. (PtJAM1.2 and PtJAZ6 co-immunoprecipitation)

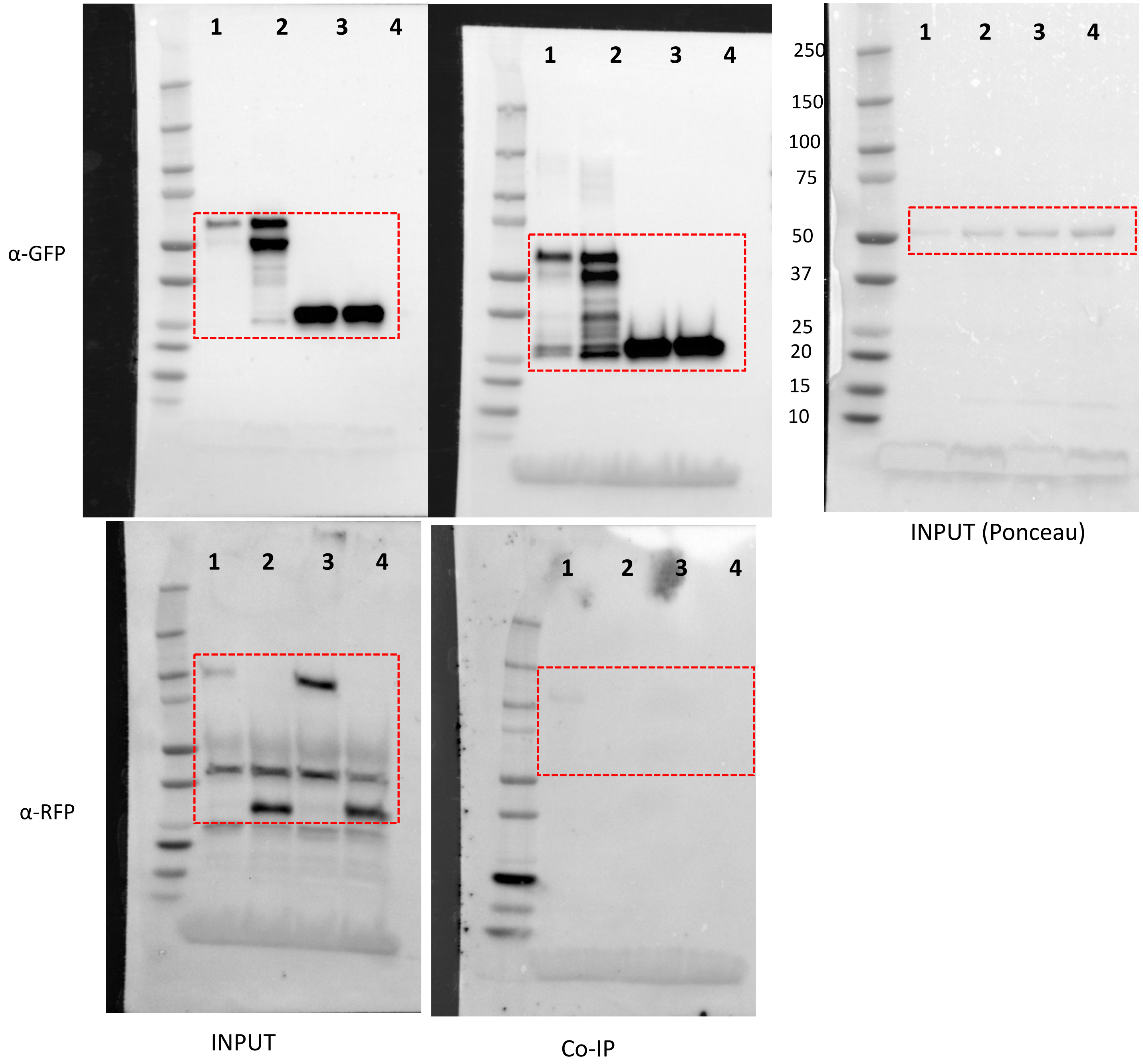

	1	2	3	4
PtJAZ6-GFP	+	+	-	-
PtJAM1.2-RFP	+	-	+	-
GFP	-	-	+	+
RFP	-	+	-	+

Full original blots cropped for Figure 1 D. (PtMYC2.1 and PtJAZ6 co-immunoprecipitation)

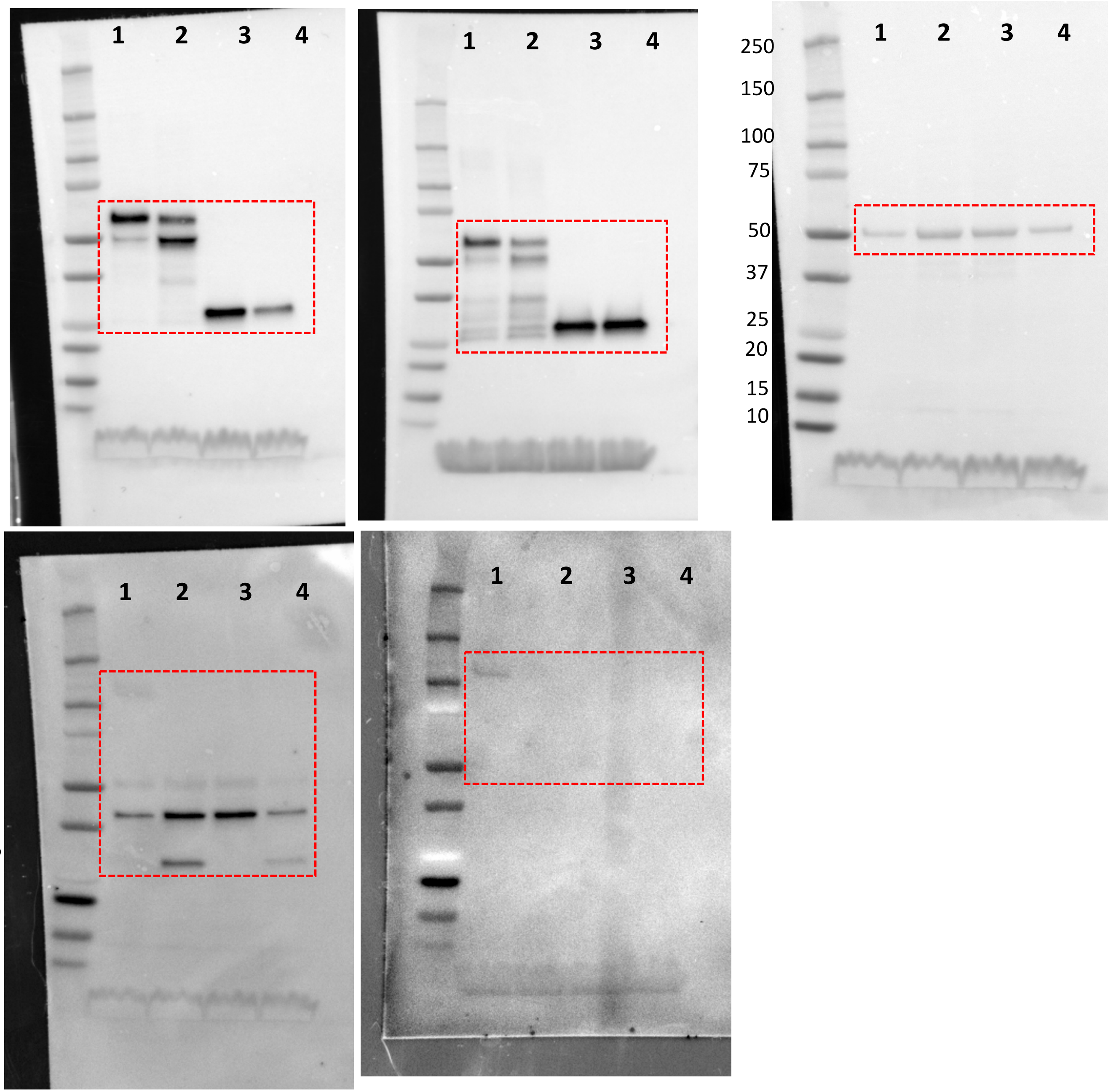

α-GFP

α-RFP

INPUT

Co-IP

	1	2	3	4
PtJAZ6-GFP	+	+	-	-
PtMYC2.1-RFP	+	-	+	-
GFP	-	-	+	+
RFP	-	+	-	+

Full original blots cropped for Figure 1 D. (PtMYC2.2 and PtJAZ6 co-immunoprecipitation)

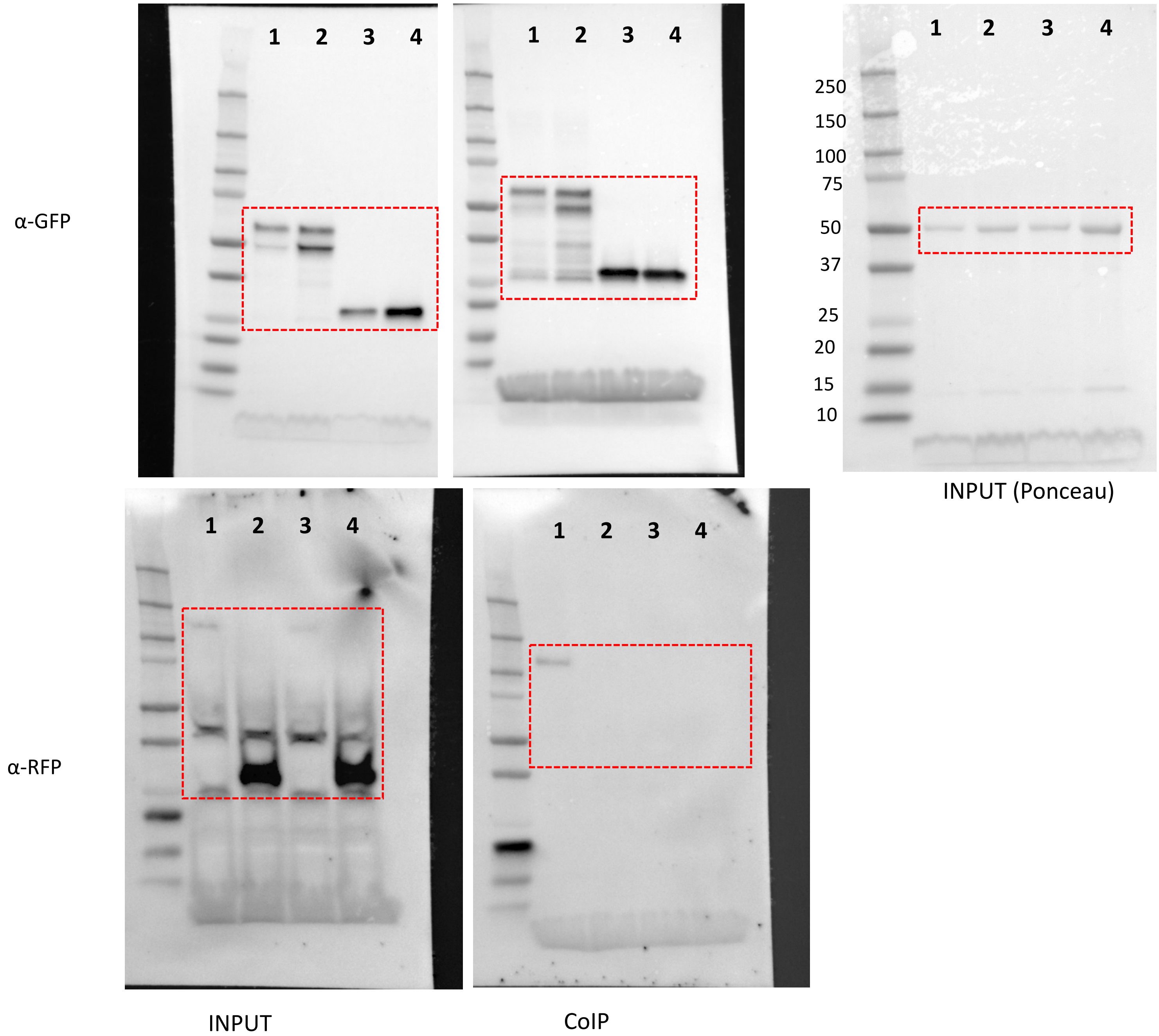

	1	2	3	4
PtJAZ6-GFP	+	+	-	-
PtMYC2.2-RFP	+	-	+	-
GFP	-	-	+	+
RFP	-	+	-	+

Pictures of β-galactosidase activity on filters used to prepare Figure 1b and 1e.

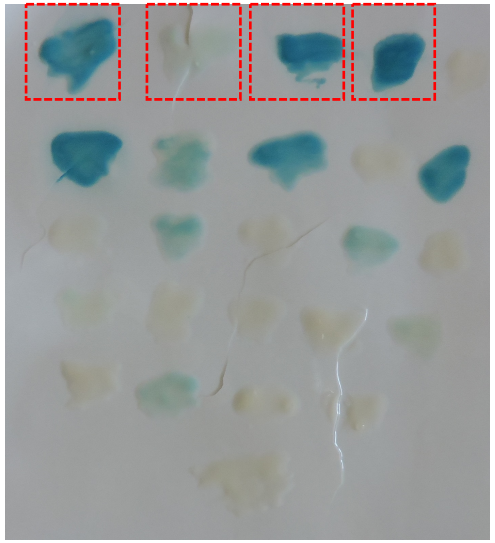

1	2	3	4	5
6	7	8	9	10
11	12	13	14	15
16	17	18	19	20
21	22	23	24	
	25			

1. PtJAZ6 – Pt JAM1.1
2. PtjAZ6 - PtJAM1.2
3. PtJAZ6 – PtMYC2.1
4. PtJAZ6- PtMYC2.2

5 to 25. Not included in this tudy

Fig.1b

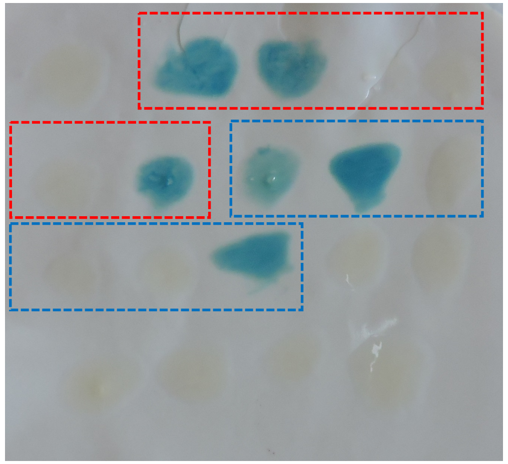

1	2	3	4	5
6	7	8	9	10
11	12	13	14	15
16	17	18.	19	

1. Not included
2. JAZ6ΔNT – PtJAM1.1
3. PtJAZ6ΔTIFY- PtJAM1.1
4. PtJAZ6Δjas- PtJAM1.1
5. PtJAZ6 NT- PtJAM1.1
6. PtJAZ6 TIFY- PtJAM1.1
7. PtJAZ6 jas - PtJAM1.1
8. JAZ6ΔNT – PtMYC2.1
9. PtJAZ6ΔTIFY- PtMYC2.1
10. PtJAZ6Δjas- PtMYC2.1
11. PtJAZ6 NT- PtMYC2.1
12. PtJAZ6 TIFY- PtMYC2.1
13. PtJAZ6 jas - PtMYC2.1

15 to 19. Not included

Fig.1e

The method we used for initial b-galactosidase activity assessment is on filters, explaining why we have to make photo mosaic (crop and rotate). At each time we have to crop, we mentioned it with white bars in the main figure and dotted lines in the supplementary info file.

Pictures of β-galactosidase activity on filters used to prepare Figure 2.

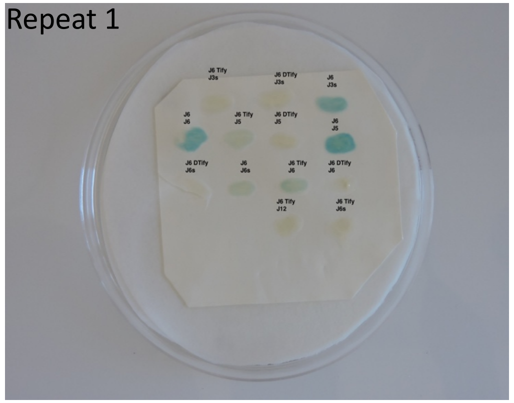

	1	2	3
4	5	6	7
8	9	10	11
		12	13

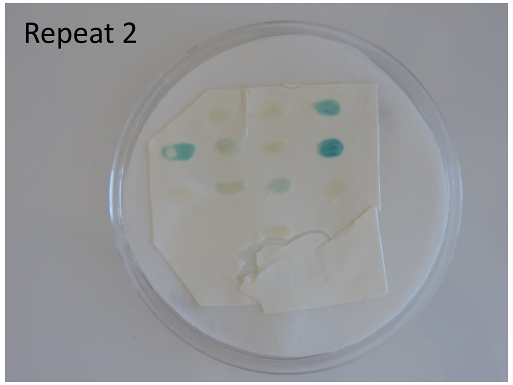

1. PtJAZ6 TIFY – PtJAZ10.2
2. JAZ6ΔTIFY – PtJAZ10.2
3. PtJAZ6 - PtJAZ10.2
4. PtJAZ6- PtJAZ6
5. PtJAZ6 TIFY- PtJAZ5
6. PtJAZ6ΔTIFY- JPtAZ5
7. PtJAZ6 - PtJAZ5
8. PtJAZ6ΔTIFY- PtJAZ1/2
9. PtJAZ6 - PtJAZ1/2
10. PtJAZ6 ΔTIFY PtJAZ6
11. PtJAZ6 TIFY-PtJAZ6
12. PtJAZ6 TIFY - PtJAZ12
13. PtJAZ6 TIFY- PtJAZ1/2

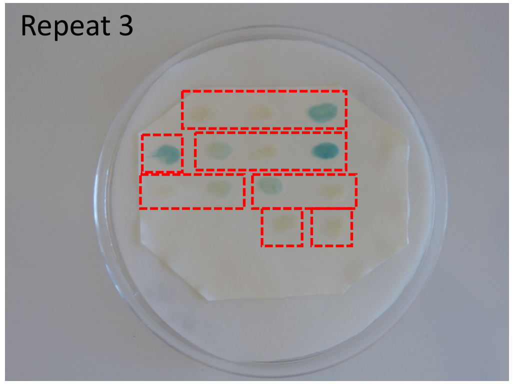

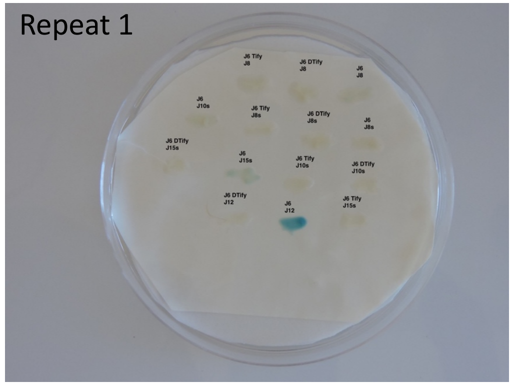

	14	15	16
17	18	19	20
21	22	23	24
	25	26	27

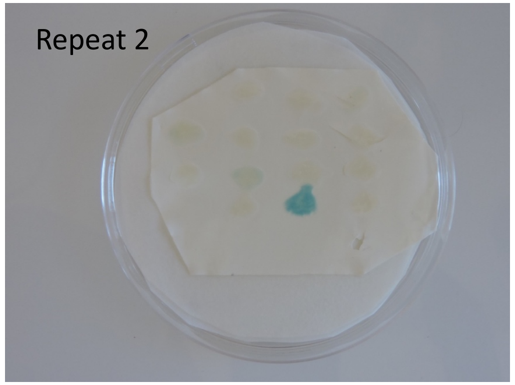

14. PtJAZ6 TIFY - PtJAZ7/8
15. PtJAZ6ΔTIFY- PtJAZ7/8
16. PtJAZ6 - PtJAZ7/8
17. PtJAZ6 - PtJAZ3.4
18. PtJAZ6 TIFY- PtJAZ3.3
19. PtJAZ6ΔTIFY- PtJAZ3.3
20. PtJAZ6 - PtJAZ3.3
21. PtJAZ6ΔTIFY- PtJAZ3.2
22. PtJAZ6 - PtJAZ3.2
23. PtJAZ6 TIFY - PtJAZ3.4
24. PtJAZ6ΔTIFY- PtJAZ3.4
25. PtJAZ6ΔTIFY- PtJAZ12
26. PtJAZ6 - PtJAZ12
27. PtJAZ6 TIFY - PtJAZ3.2

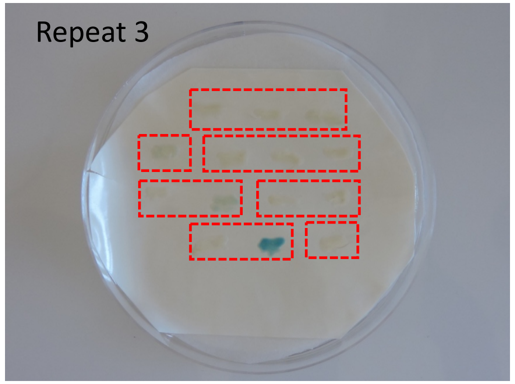

Images of three biological repeats for X-Gal assays. One of the three biological repeat was chosen for illustration. The method we used for initial b-galactosidase activity assessment is on filters, explaining why we have to make photo mosaic (crop and rotate). At each time we have to crop, we mentioned it with white bars in the main figure and red lines in the supplementary info file.

Pictures of β-galactosidase activity on filters used to prepare Figure 4a and Figure 1b,e.

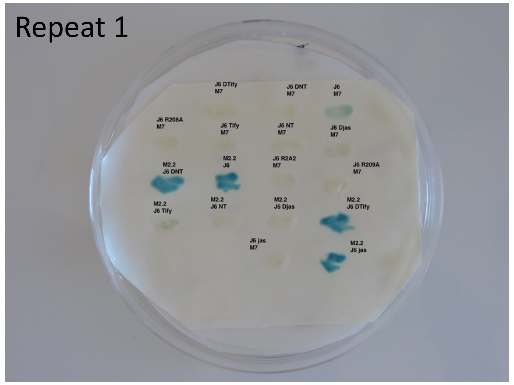

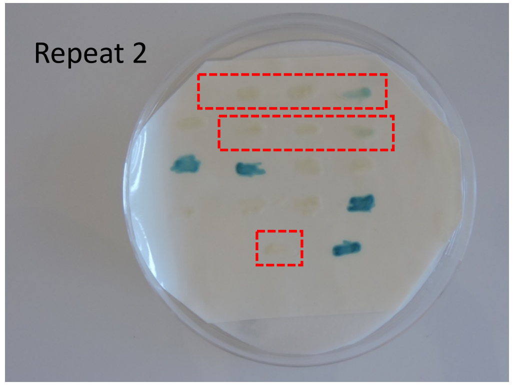

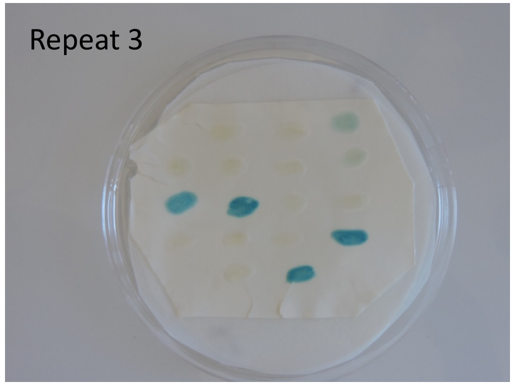

	1	2	3
4	5	6	7
8	9	10	11
12	13	14	15
		16	17

1. PtJAZ6ΔTIFY – MiSSP7
2. PtJAZ6ΔNT – MiSSP7
3. PtJAZ6 – MiSSP7

Fig. 4a

4. PtJAZ6R208A – MiSSP7
5. PtJAZ6 TIFY – MiSSP7
6. PtJAZ6 NT – MiSSP7
7. PtJAZ6Δjas – MiSSP7

Fig. 4a

8. PtJAZ6ΔNT - PtMYC2.2
9. PtJAZ6 – PtMYC2.2
10. Not included in the study
11. Not included in the study
12. PtJAZ6 TIFY – PtMYC2.2
13. PtJAZ6 NT – PtMYC2.2
14. PtJAZ6Δjas – PtMYC2.2
15. PtJAZ6ΔTIFY – PtMYC2.2
16. PtJAZ6 jas – MiSSP7

Fig. 4a

17. PtJAZ6 jas – PtMYC2.2

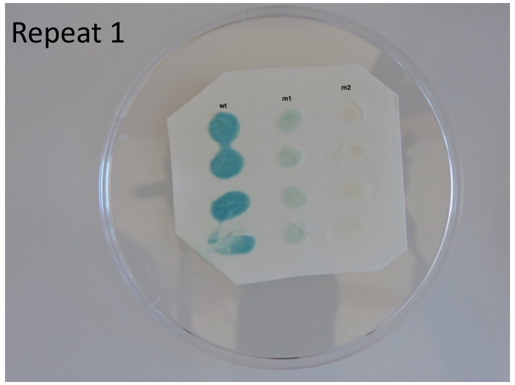

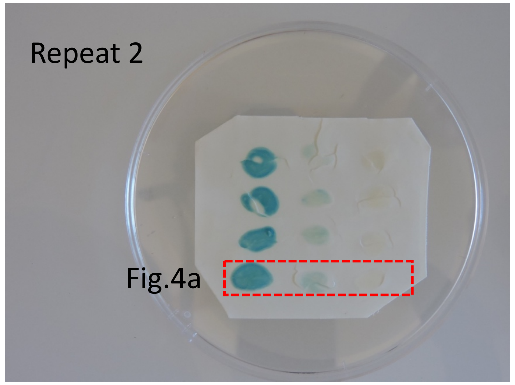

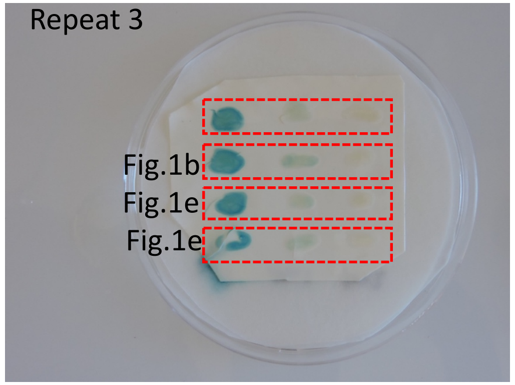

1	2	3
1	2	3
1	2	3
1	2	3

1. WT: strong interaction control
2. m1: weak interaction control
3. M2: no interaction

Images of three biological repeats for X-Gal assays. One of the three biological repeat was chosen for illustration. The method we used for initial b-galactosidase activity assessment is on filters, explaining why we have to make photo mosaic (crop and rotate). At each time we have to crop, we mentioned it with white bars in the main figure and red lines in the supplementary info file.

Pictures of β-galactosidase activity on filters used to prepare Figure 4b

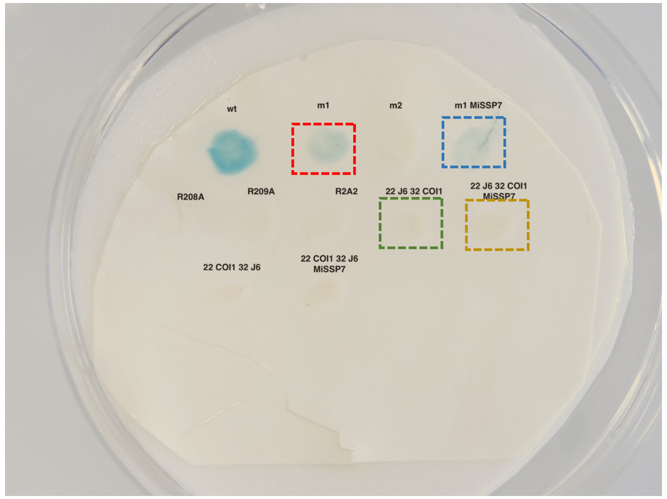

1	2	3	4	
5	6	7	8	9
10	11			

1. WT: strong interaction control
2. m1: weak interaction control
3. m2: no interaction
4. m1 + MiSSP7
5. Not included in this paper
6. Not included in this paper
7. Not included in this paper
8. AD:PtJAZ6 /DBD:PtCOI1
9. AD:PtJAZ6 /DBD:PtCOI1 + MiSSP7
10. Not included in this paper
11. Not included in this paper

+ coronatine (COR)

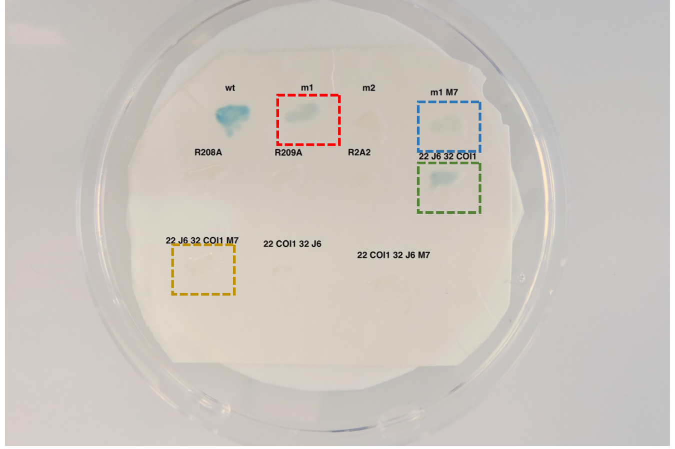

1	2	3	4
5	6	7	8
9	10	11	

Images of three biological repeats for X-Gal assays. One of the three biological repeat was chosen for illustration. The method we used for initial b-galactosidase activity assessment is on filters, explaining why we have to make photo mosaic (crop and rotate). At each time we have to crop, we mentioned it with white bars in the main figure and red lines in the supplementary info file.

Pictures of β-galactosidase activity on filters used to prepare Figure 3d.

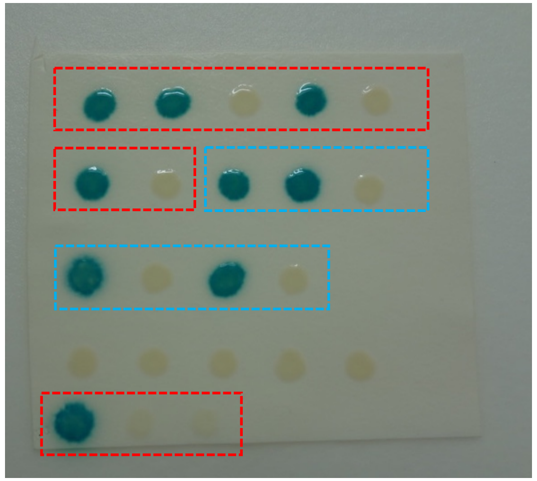

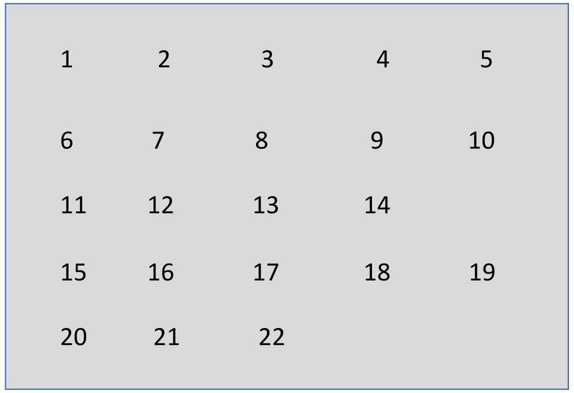

1. DBD:PtNINJA1/ AD:PtJAZ6
2. DBD:PtNINJA1/ AD:PtJAZ6 ΔNT
3. DBD:PtNINJA1/ AD:PtJAZ6 ΔTIFY
4. DBD:PtNINJA1/ AD:PtJAZ6 ΔJAS
5. DBD:PtNINJA1/ AD:PtJAZ6 NT
6. DBD:PtNINJA1/ AD:PtJAZ6 TIFY
7. DBD:PtNINJA1/ AD:PtJAZ6 JAS
8. DBD:PtNINJA3/ AD:PtJAZ6
9. DBD:PtNINJA3/ AD:PtJAZ6 ΔNT
10. DBD:PtNINJA3/ AD:PtJAZ6 ΔTIFY
11. DBD:PtNINJA3/ AD:PtJAZ6 ΔJAS
12. DBD:PtNINJA3/ AD:PtJAZ6 NT
13. DBD:PtNINJA3/ AD:PtJAZ6 TIFY
14. DBD:PtNINJA3/ AD:PtJAZ6 JAS
15. DBD:LbMiSSP7/ AD:PtMYC2.1
16. DBD:LbMiSSP7/ AD:PtMYC2.2
17. DBD:LbMiSSP7/ AD:PtMYC2.1
18. DBD:LbMiSSP7/ AD:PtMYC2.2
19. DBD:PtJAZ6
20. +++ (strong interaction control)
21. + (weak interaction control)
22. – (no interaction)

Pictures of β-galactosidase activity on filters used to prepare Figure 3c

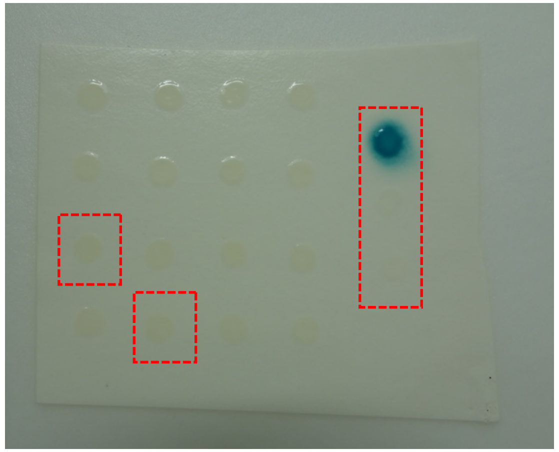

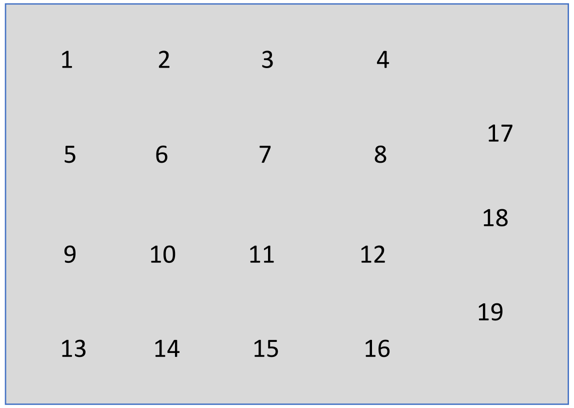

1. Not included in this manuscript
2. Not included in this manuscript
3. Not included in this manuscript
4. Not included in this manuscript
5. Not included in this manuscript
6. Not included in this manuscript
7. Not included in this manuscript
8. Not included in this manuscript
9. DBD:PtTPR4.1/ AD:PtJAZ6
10. DBD:PtTPR4.1/ AD:PtJAZ6
11. Not included in this manuscript
12. Not included in this manuscript
13. DBD:PtTPR4.1/ AD:PtJAZ6 EARm
14. DBD:PtTPR4.1/ AD:PtJAZ6 EARm
15. Not included in this manuscript
16. Not included in this manuscript
17. +++
18. +
19. -

Images of three biological repeats for X-Gal assays. One of the three biological repeat was chosen for illustration. The method we used for initial b-galactosidase activity assessment is on filters, explaining why we have to make photo mosaic (crop and rotate). At each time we have to crop, we mentioned it with white bars in the main figure and red lines in the supplementary info file.

Pictures of β-galactosidase activity on filters used to prepare Figure 5 and S5.

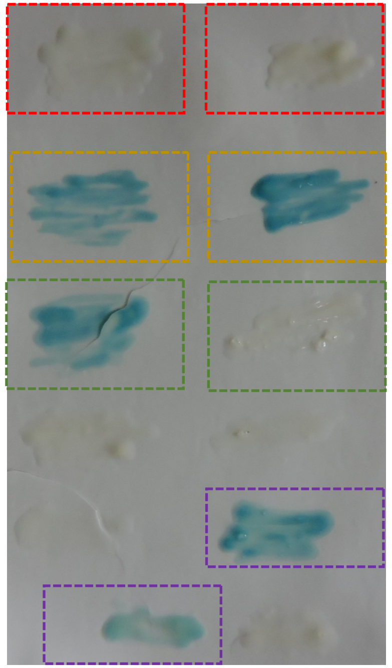

1	1+ MiSSP7
2	2+MiSSP7
3	3+MiSSP7
4	5 + MiSSP7
5	6+MiSSP7
6	4+MiSSP7

1. m1: weak interaction control
2. PtMYC2.1-PtJAZ6
3. PtMYC2.2-PtJAZ6
4. Not included in this paper
5. Not included in this paper
6. PtJAZ10.2-PtjAZ6

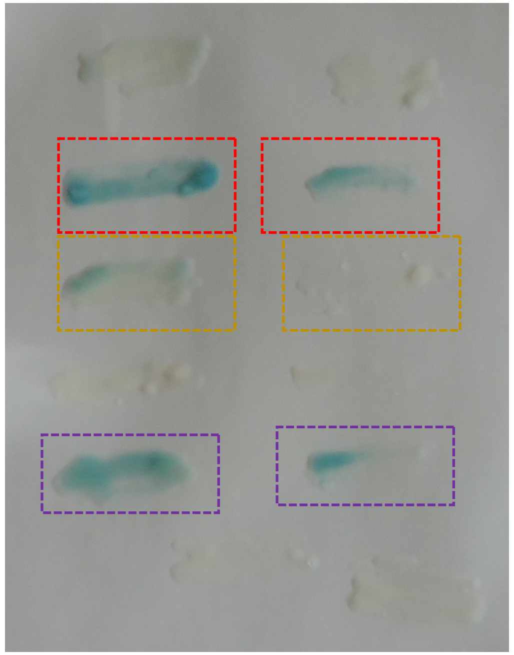

1	1+ MiSSP7
2	2+MiSSP7
3	3+MiSSP7
4	4 + MiSSP7
5	5+MiSSP7
6	6+MiSSP7

1. Pt1s1-PtJAZ6
2. PtJAZ5-PtJAZ6
3. PtJAZ6-PtJAZ6
4. Not included in this paper
5. PtNINJA3-PtJAZ6
6. PtJAZ3.1-PtJAZ6

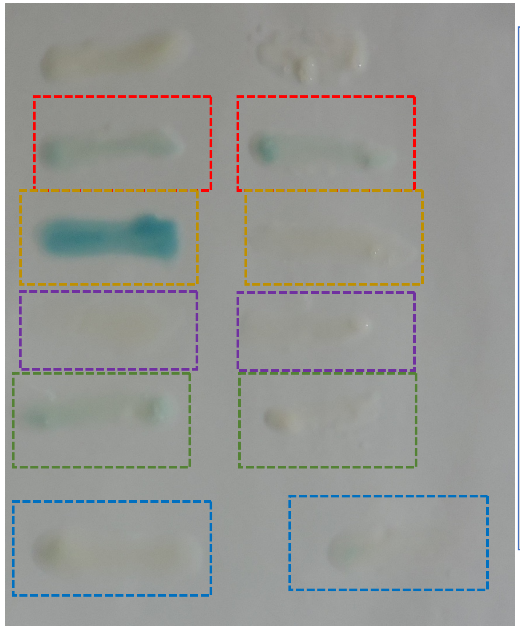

1	1+ MiSSP7
2	2+MiSSP7
3	3+MiSSP7
4	4 + MiSSP7
5	5+MiSSP7
6	6+MiSSP7

1. Not included in this paper
2. PtJAZ1/2-PtJAZ6
3. PtJAZ12-PtJAZ6
4. PtJAZ3.3-PtJAZ6
5. PtJAZ3.2-PtJAZ6
6. PtJAZ7/8-PtJAZ6

Images of three biological repeats for X-Gal assays. One of the three biological repeat was chosen for illustration. The method we used for initial b-galactosidase activity assessment is on filters, explaining why we have to make photo mosaic (crop and rotate). At each time we have to crop, we mentioned it with white bars in the main figure and red lines in the supplementary info file.
